# *RePhine*: An Integrative Method for Identification of Drug Response-related Transcriptional Regulators

**DOI:** 10.1016/j.gpb.2019.09.008

**Published:** 2021-03-10

**Authors:** Xujun Wang, Zhengtao Zhang, Wenyi Qin, Shiyi Liu, Cong Liu, Georgi Z. Genchev, Lijian Hui, Hongyu Zhao, Hui Lu

**Affiliations:** 1 SJTU-Yale Joint Center for Biostatistics and Data Science, Department of Bioinformatics and Biostatistics, School of Life Science and Biotechnology, Shanghai Jiao Tong University, Shanghai 200240, China; 2 State Key Laboratory of Cell Biology, CAS Center for Excellence in Molecular Cell Science, Shanghai Institute of Biochemistry and Cell Biology, Shanghai 200031, China; 3 Department of Bioengineering, University of Illinois at Chicago, Chicago, IL 60607, USA; 4 Department of Genetics, School of Medicine, Yale University, New Haven, CT 06511, USA; 5 Department of Medical Informatics, Columbia University, New York, NY 10032, USA; 6 Bulgarian Institute for Genomics and Precision Medicine, Sofia 1000, Bulgaria; 7 Department of Biostatistics, Yale School of Public Health, New Haven, CT 06511, USA; 8 Institute of Science and Technology for Brain-Inspired Intelligence, Fudan University, Shanghai 200433, China; 9 Center for Biomedical Informatics, Shanghai Children’s Hospital, Shanghai 200040, China

**Keywords:** Pharmacogenomics, ChIP-seq, Transcriptional regulator, BRAF inhibitor resistance, Drug resistance

## Abstract

**Transcriptional regulators** (TRs) participate in essential processes in cancer pathogenesis and are critical therapeutic targets. Identification of drug response-related TRs from cell line-based compound screening data is often challenging due to low mRNA abundance of TRs, protein modifications, and other confounders (CFs). In this study, we developed a regression-based pharmacogenomic and **ChIP-seq** data integration method (*RePhine*) to infer the impact of TRs on drug response through integrative analyses of pharmacogenomic and ChIP-seq data. *RePhine* was evaluated in simulation and pharmacogenomic data and was applied to pan-cancer datasets with the goal of biological discovery. In simulation data with added noises or CFs and in pharmacogenomic data, *RePhine* demonstrated an improved performance in comparison with three commonly used methods (including Pearson correlation analysis, logistic regression model, and gene set enrichment analysis). Utilizing *RePhine* and Cancer Cell Line Encyclopedia data, we observed that *RePhine*-derived TR signatures could effectively cluster drugs with different mechanisms of action. *RePhine* predicted that loss-of-function of EZH2/PRC2 reduces cancer cell sensitivity toward the BRAF inhibitor PLX4720. Experimental validation confirmed that pharmacological EZH2 inhibition increases the resistance of cancer cells to PLX4720 treatment. Our results support that *RePhine* is a useful tool for inferring drug response-related TRs and for potential therapeutic applications. The source code for *RePhine* is freely available at https://github.com/coexps/RePhine.

## Introduction

Cancer, one of the most common causes of death [1], is characterized by uncontrolled cell division [2], [3]. Pharmacological treatments such as chemotherapy, targeted therapy, and immunotherapy are widely used. Accurate drug selection has the potential to improve patient outcomes by matching the patient’s genomic characteristics with the most effective treatment available [4]. The comprehensive delineation of the association between drug response and omics features by using a suitable computational model may significantly contribute to preclinical research and drive clinical decision-making. Most existing drug response prediction methods are based on direct putative correlations between individual genes’ mRNA levels and drug sensitivity measurement [4]. Such predictions, however, are not always sufficiently robust due to the inherent experimental noises [5], [6]. The robust identification of drug response biomarkers remains a significant challenge.

Many transcriptional regulators (TRs), including transcription factors (TFs) and chromatin regulators (CRs), can control cell development and cell survival by regulating the expression of target genes [7], [8], [9]. For example, inhibiting the TFs ESR1 and SP1 could induce the death of myeloma cells [10] and targeting the CR BRD4 rescues the response to the γ-secretase inhibitors in T-cell acute lymphoblastic leukemia [11]. These studies reveal the complicated and vital roles of TRs and emphasize the necessity of systematic identification of drug response-related TRs. However, a number of biological features of TRs (*e.g.*, low mRNA abundance, protein modifications, and localizations) pose challenges to such identification [12], [13], [14]. For example, by using Cancer Cell Line Encyclopedia (CCLE) data, the resistance role of zinc finger E-box binding homeobox 1 (ZEB1) can be directly reflected by its mRNA level correlated to the response to the drug erlotinib, but there is no obvious correlation in the case of Fos proto-oncogene (FOS) (Figure S1A and B), though both TRs are known biomarkers of erlotinib [15], [16]. Therefore, solely relying on the mRNA levels of TRs to identify TR-drug response relationship lacks identification power (false negatives) and may even lead to inaccurate associations (false positives) in the existing compound screening data. Given the importance of TRs in tumorigenesis and the limitations of existing data, a new strategy is required to explore the functional roles of TRs in drug response [17]. However, the effects of copy number variations (CNVs), data noises, and confounding mutations of some kinase genes or tumor suppressor genes, as well as how to accurately infer targets, pose challenges in this identification effort.

**Figure 1:**
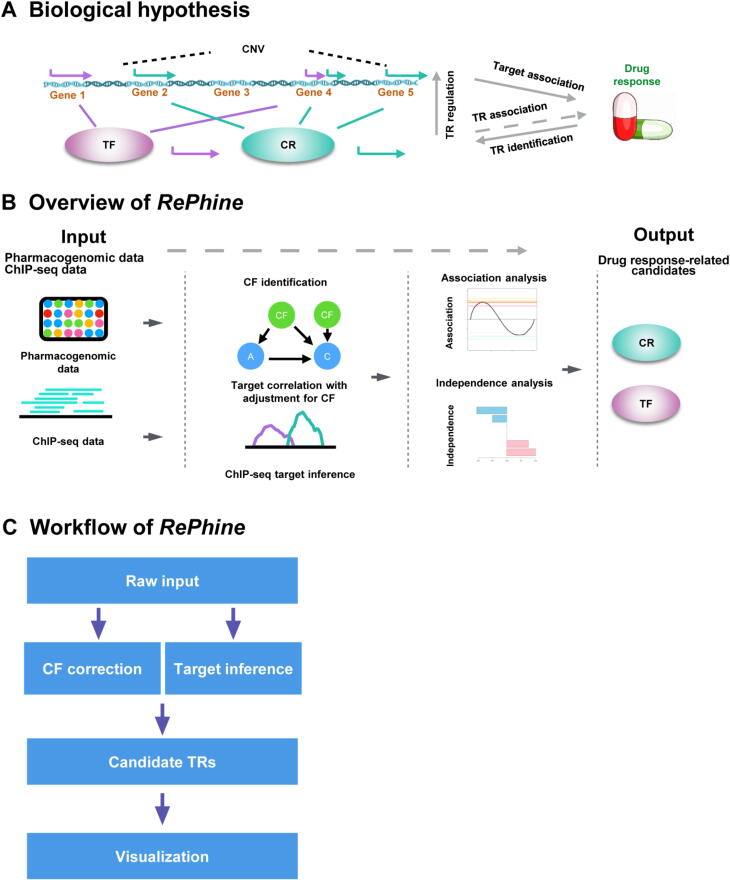
**A schematic view of *RePhine* hypothesis and analysis workflow. A.** The biological hypothesis of *RePhine* model. Association between a TR and drug response can be inferred by the pattern of its targets. TR identification is based on the TR association with drug response, which is indirect information (indicated by the grey dashed arrow) that can be inferred from TR regulation profiles and target association with drug response. **B.** Overview of the *RePhine* analysis procedure. The purpose of *RePhine* is to identify drug response-related TRs by integrating pharmacogenomic data and ChIP-seq data. **C.***RePhine* analysis workflow. TR, transcriptional regulator; TF, transcription factor; CR, chromatin regulator; CNV, copy number variation; CF, confounder; A represents a target gene of the TR; C represents drug response.

In this study, we hypothesized that the association of a TR with drug response could also be inferred through the association between the expression of its downstream targets and the drug treatment (Figure 1**A**). Based on this notion, we developed the regression-based pharmacogenomic and ChIP-seq data integration method (*RePhine*) which takes pharmacogenomic and ChIP-seq data as input and identifies TRs associated with response to pharmacological drug therapy (Figure 1B). We demonstrated that *RePhine* has an improved performance in comparison with several commonly used methods in TR identification in simulation and pharmacogenomic data. Through the *RePhine* analysis pipeline, we characterized TR response signatures of drug treatments. Utilizing *RePhine*, we uncovered a BRAF inhibitor resistance mechanism relevant to the functional loss of polycomb repressive complex 2 (PRC2). This mechanism was then validated by drug treatment experiments in cell lines and bioinformatic analyses in CRISPR screening data, The Cancer Genome Atlas (TCGA) patient cohorts, and differentially expressed genes between BRAF inhibitor-sensitive and resistant cells.

## Method

### Description of the *RePhine* method

The *RePhine* method is designed to identify the TRs whose targets have concordant correlations with drug response (Figure 1B and C). *RePhine* adjusts for confounders (CFs) by calculating the partial correlation coefficients between expression of all the genes and one specific drug treatment across all the cell lines. Next, *RePhine* measures the TR targets quantitatively from ChIP-seq data. Then, *RePhine* identifies the drug response-related TRs by regressing the TR target measurements on the partial correlation coefficients. *RePhine* employs the two metrics — univariate *P* value (uni*P*) and multivariate *P* value (multi*P*) — to evaluate the significance of the associations of TRs to the given drug.

The *RePhine* method consists of the four steps that were described as below (Figure 1C, Figure S2; File S1).

## Step 1: CF identification and correction

In Step 1.1, the effect of CNVs is adjusted. To account for the fact that gene copy numbers can influence gene expression levels independent of TR regulation [18], *RePhine* utilizes a linear model to evaluate the impact of a TR on the expression of its targets.

The following model is fitted:

(1) $$Y_{n}^{\mathrm{expr}}=\beta^{cnv}x_{n}^{\mathrm{cnv}}+\varepsilon_{n}^{adj\_expr}$$

where $x_{n}^{\mathrm{cnv}}$ is the copy number of a certain gene in the cell line *n*; $Y_{n}^{\mathrm{expr}}$ is the expression value of this gene in the same cell line; the fitted residual $\varepsilon_{n}^{adj\_expr}$ is considered as the adjusted expression; $\beta^{cnv}$ is the coefficient.

In step 1.2, mutation CFs are identified. Some somatic mutations can influence drug response independent of TR regulation, and such mutations may confound the identification of drug response-related TRs. To identify such mutations related to drug response, a two-stage procedure is applied.

In step 1.2.1, genes with mutations at extremely low frequency across the cancer cell lines (<5% of the number of the cell lines) are filtered.

In step 1.2.2, Adaptive Lasso (AL) is then used to select significantly drug response-related mutated genes [19]:

(2) $$\beta^{\mathrm{Adaptive}-LASSO}=\arg\min_{\beta }{\|y^{\mathrm{drug}}-\sum_{j=1}^{M}x_{j}^{\mathrm{mut}}\beta_{j}^{\mathrm{mut}}\|}^{2}+\lambda \sum_{j=1}^{M}\left|\beta_{j}^{\mathrm{mut}}\right|\tau_{j}$$

where $x_{j}^{\mathrm{mut}}$ = ($x_{1j}^{\mathrm{mut}},\cdots ,x_{\mathrm{nj}}^{\mathrm{mut}}$)^T^ represents the mutation status (0 for the wild type and 1 for the mutation) in the gene *j* (from the step 1.2.1) across all the *n* cell lines; $y^{\mathrm{drug}}$= ($y_{1}^{\mathrm{drug}},\cdots ,y_{n}^{\mathrm{drug}}$)^T^ represents the drug sensitivity score in the corresponding cell lines; $\tau_{j}=1/\left|\tilde{\beta_{j}}\right|$; $\tilde{\beta }={\left(\tilde{\beta_{1}},\cdots ,\tilde{\beta_{j}},\cdots ,\tilde{\beta_{M}}\right)}^{T}$ is the ordinary least-squares estimator; λ is selected by leave-one-out cross-validation; *M* is the gene count from step 1.2.1. $\beta^{\mathrm{mut}}$ is the coefficient estimated by the equation.

The R “*glmnet*” package is utilized for step 1.2.2. A likelihood ratio test is used to estimate the significance of β. Genes with both significant uni*P* and multi*P* (*P* < 0.01) are used as the CFs in the subsequent partial correlation computation step 1.3.

In step 1.3, the partial correlation coefficients are calculated to adjust the effect of the CFs (estimated from step 1.2) and accurately measure the association between the drug response and the expression of all the genes (estimated from step 1.1). The detailed calculation of the partial correlation coefficients is described in File S1. The R “*ppcor*” package is used to perform the computation for each gene in this step.

In step 1.4, the partial correlation coefficients of cancer type-specific genes are down weighted. Expression in tumor *vs.* normal samples in 14 cancer types, sourced from TCGA data, is compared before this step. Then *RePhine* performs down-weighting of the genes which are differentially expressed [calculated by “*limma*” package with false discovery rate (FDR) < 0.001] in less than 1/3 of the cancer types. Then, *RePhine* is applied to a pan-cancer dataset such as CCLE, aiming to identify pan-cancer drug response-related TRs. The goal of this step is to reduce the effect of cancer type-specific genes. For such genes, the partial correlation coefficients are set to zero. In our calculation, only 10% of the genes are corrected in this step and most TRs are not affected (Table S1). Compared to the non-filtering procedure, the filtering step up-weights the TRs related to the common cancer pathways as expected (Table S1). This step is optional and it can be skipped when applying *RePhine* to other datasets.

## Step 2: Regulatory potential calculation

To determine the targets of TRs from ChIP-seq data, we implement a modified model [20] by additionally considering peak signal strength; in the original model, only peak counts and distances to genes were considered. Regulatory potential (RP) scores are calculated for given genes to measure the regulation strength of TRs. The implemented model is:

(3) $${\mathrm{RP}}_{\mathrm{gene}}^{\mathrm{TR}}=\sum_{i=1}^{k}\left(e^{-(0.5+4d_{i})}\times m_{i}\right)$$

where *k* represents the count of peaks around the gene; *d* is the distance from the center of peak *i* to the transcriptional start site (TSS) of the gene; $e^{-(0.5+4d_{i})}$ is defined in the original study [20]; *m* is the overall enrichment of the signal for the peak regions from the Encyclopedia of DNA Elements (ENCODE) database.

## Step 3: Selection of the TRs most relevant to drug response

In step 3.1, TR independence is estimated through Elastic-Net (EN) model. Many TRs have overlapping regulation effects with other TRs. To select the key regulators among the overlapping TRs, an EN method is conducted by regressing the RP scores on the partial correlations of all the genes. By using this step, *RePhine* takes both effects of TFs and CRs into account simultaneously. The EN estimator [21] is defined as:

(4) $${\hat{\beta }}_{\alpha ,\lambda }=\arg\min_{\beta }\left\{{\|y^{\mathrm{partial}}-\sum_{t=1}^{p}x_{t}^{\mathrm{RP}}\beta_{t}^{\mathrm{RP}}\|}^{2}+\lambda \left[\left(1-\alpha \right)\sum_{t=1}^{p}{\beta_{t}^{\mathrm{RP}}}^{2}+\alpha \sum_{t=1}^{p}\left|\beta_{t}^{\mathrm{RP}}\right|\right]\right\}$$

where $x_{t}^{\mathrm{RP}}={\left(x_{1t}^{\mathrm{RP}},\cdots ,x_{\mathrm{nt}}^{\mathrm{RP}}\right)}^{T}$is a vector of gene RP scores corresponding to the *t*-th TR (TR_t_) from step 2; *p* is the count of TRs; $y^{\mathrm{partial}}$ represents the partial correlation of the corresponding gene generated in step 1.4.

If multiple replicates or samples in different cell lines are available for the same TR_t_, *RePhine* only chooses the replicate or sample with the largest statistical effect of association between the targets and the given drug (the most significant uni*P*) to represent the TR_t_ (analogously to the approach of the RABIT method [17]).

To determine the α in the EN model, we tried different values of α and found highly consistent results of TR selection (see File S1 and Table S2 for more details). Therefore, α is fixed to 0.8 to shorten the time of calculations. λ is obtained from leave-one-out cross-validation where the parameter “lambda.1se” is used to avoid the over-fitting. The R “*glmnet*” package is used in the calculation.

In step 3.2, a likelihood ratio test is utilized to assess the significance of estimated coefficients for both the EN model step (step 3.1) and the AL model step (step 1.2.2); uni*P* and multi*P* are calculated by comparing the goodness of fit of these two models with inclusion/exclusion of the selected variable. In the univariate setting, model 1: y=βx+intercept is compared to model 2: y=intercept. In multivariate setting, all predictors selected from AL or EN are regarded as “S”; thus, model 1: y=βS+intercept is compared to model 2: $y=\beta S_{-x}+intercept$, where *×* is the variable of interest. Variables *y* and *×* in the AL and EN models are the same as those in the step 1.2.2 and step 3.1, respectively. The R “*lrtest*” package is used in this step.

In these procedures, uni*P* is used to measure the overall significance of associations between RP scores and partial correlation coefficients; multi*P* is applied to assess the independence of the associations.

## Step 4: Visualization of the association

In this step, an enrichment algorithm is additionally implemented to visualize the association and the enrichment patterns. Permutation *P* value (permu*P*) is used to assess significance of the enrichment. The details of the algorithm are as follows:

1) Rank the *N* genes according to the partial correlation coefficients.

2) Normalize the vector $P^{\mathrm{reg}}$ (the RP scores) to a range [0, 1] across the genes within the sample by dividing by the maximum values.

3) Evaluate values for the genes as belonging (hit) and not belonging (miss) to the TR targets weighted by the partial correlation $r^{\mathrm{partial}}$. For the top *i* genes ranked by partial correlations:

(5) $$D_{\mathrm{hit}}\left(TR,i\right)=\sum_{j\leq i}P_{j}^{\mathrm{reg}}\times \frac{{\left|r_{j}^{\mathrm{partial}}\right|}^{k}}{N_{R}}$$

where $N_{R}=\sum_{j=1}^{N}P_{j}^{\mathrm{reg}}\times {\left|r_{j}^{\mathrm{partial}}\right|}^{k}$and *k* = 1.

(6) $$D_{\mathrm{miss}}\left(TR,i\right)=\sum_{j\leq i}\left(1-P_{j}^{\mathrm{reg}}\right)\times \frac{1}{N-\sum_{j=1}^{N}P_{j}^{\mathrm{reg}}}$$

where *N* represents the gene count.

4) Estimate the maximum deviation of *D_hit_* − *D_miss_* from zero. For random distributions of TR targets, the enrichment score ES(TR) will be a relatively small value. In contrast, if targets with higher RP scores assemble at the top or bottom of the partial correlation list, the ES(TR) will be high. When $P^{\mathrm{reg}}$ is binary (0 or 1), ES(TR) reduces to the original gene set enrichment analysis (GSEA) ES (see File S1 for details). When $P^{\mathrm{reg}}$ is equal to 0 or 1 and *k* is simultaneously equal to 0, the ES(TR) will be the standard Kolmogorov-Smirnov test statistics [22].

5) Permute the gene labels and re-compute the ESs. Repeat 1000 times to obtain the distribution of ES_random_. The assessment of *P* value is based on the positive or negative segments of the distribution of ES_random_ depending on the sign of ES(TR).

Pharmacologically-relevant patterns such as positive-association, negative-association, and non-association could be distinguished and visualized through the aforementioned formula (Figure S3).

### Drug combination screening

The interaction between PLX4720 and GSK126 is measured by the combination index (CI) derived from CalcuSyn [23]. CI = 1 indicate additive effects; CI > 1 and CI < 1 indicate antagonism and synergism, respectively.

## Results

### *RePhine* has an improved performance in simulation data with added noises and CFs

To systematically examine whether *RePhine* can accurately identify the associations between the TRs and the drug response, its performance was evaluated in the simulation datasets. We compared *RePhine* with three commonly used methods: Pearson correlation (PC) analysis, logistic regression model, and GSEA (Figure S4). With the increase of CF counts and expression noise levels, *RePhine* showed a significantly improved performance (Figures S4A–C). Although *RePhine* didn’t overperformed GSEA with the expression noise level increasing because both methods consider target information, *RePhine* had an improved performance when noise was added to the RP scores of target information (Figures S4D–G). In addition, we also compared these methods in the scenario where multiple noises and CFs were added simultaneously to a balanced dataset and an imbalanced dataset, respectively (Figure S4H and I). *RePhine* exhibited higher area under the receiver operating characteristic (AUROC) values than the three commonly used methods in the balanced dataset when noise level and CF count increasing. Similarly, *RePhine* showed higher area under the precision recall curve (AUPRC) values in the imbalanced dataset when noise level and CF count increasing. These simulation results clearly demonstrate the advantage of *RePhine* over these commonly used methods (for more details of the comparison, see File S1). *RePhine* employs a novel strategy with careful consideration of noises and CFs and enables robust identification of TRs.

### *RePhine* enables identification of TRs with significant and non-significant correlation between their mRNA levels and drug response

**Figure 2:**
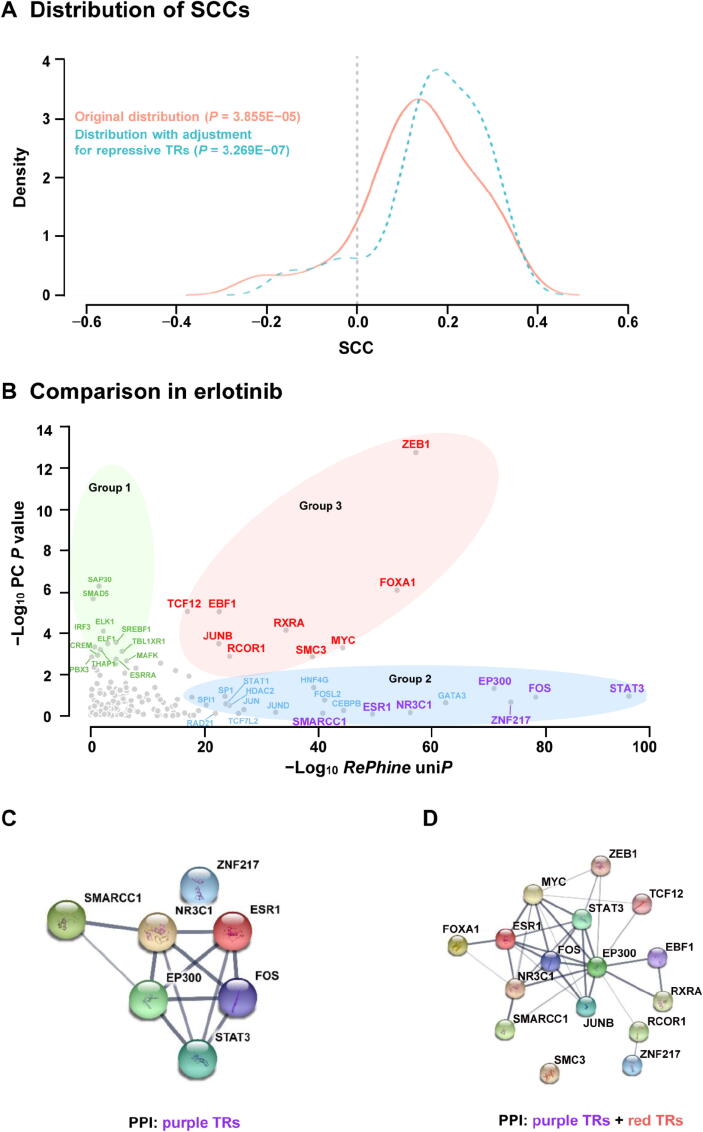
**Comparison of the results between *RePhine* and PC analysis in CCLE data. A.** Density plot for distribution of SCCs between *RePhine* significant scores (−Log_10_*RePhine* uni*P* with direction) and PCCs of all 24 CCLE drugs. PCCs are calculated between TR mRNA level and drug response. **B.** Comparison of *RePhine* and PC in erlotinib. TRs shown in green (PC-correlated-only), blue and purple (*RePhine*-correlated-only), and red (PC-*RePhine*-shared-correlated) represent candidates that have significant *P* values in PC analysis, *RePhine*, and both methods, respectively. TRs shown in purple represent *RePhine*-correlated-only independent TRs that additionally have *RePhine* significant multi*P* (within the *RePhine* multivariate EN cutoff multi*P* < 0.005). **C.** PPI network among *RePhine*-correlated-only independent TRs. **D.** PPI network by pooling *RePhine*-correlated-only independent TRs and PC-*RePhine*-shared-correlated TRs together. PC, Pearson correlation; CCLE, Cancer Cell Line Encyclopedia; SCC, Spearman correlation coefficient; PCC, Pearson correlation coefficient; uni*P*, univariate *P* value; multi*P*, multivariate *P* value; EN, Elastic-Net; PPI, protein–protein interaction.

When applied to pharmacogenomic data, *RePhine* can identify TRs which have significant correlation between their mRNA expression levels and drug response. Furthermore, *RePhine* can also identify candidate TRs which do not have significant correlation between their mRNA expression levels and drug response (Figure 2**A and B**). We applied *RePhine* to data from CCLE and ENCODE and performed both *RePhine* and PC analysis to compare the two methods which are based on two different biological assumptions. For each TR, two metrics were calculated: 1) *RePhine* significant score (uni*P*) in log scale with direction (resistance or sensitivity) and 2) Pearson correlation coefficient (PCC) between its mRNA level and drug response. For the 24 drugs available in CCLE, the two metrics were positively correlated in most drugs (Figure 2A, Figure S5A), supporting the biological assumption that TRs influencing the target regulation by their mRNA activation or deficiency could also be effectively identified by target-based methods such as *RePhine*. In the specific examples of erlotinib (a well-established anti-cancer targeted therapy drug agent [15]) and paclitaxel (a chemotherapy drug), *RePhine* significant scores (uni*P*) were highly correlated with the PCCs across all the TRs (Figure S5B and C).

How does *RePhine* perform on TRs which do not have significant PC between their mRNA expression levels and drug response? We then chose erlotinib to assess this issue. First, we divided the TRs into 3 groups. Group 1 contained the PC-correlated-only TRs (Figure 2B, green set; having significant PC between their mRNA expression levels and drug response but not *RePhine* significant uni*P*). Group 2 contained the *RePhine*-correlated-only TRs (Figure 2B, blue and purple sets; having *RePhine* significant uni*P* between their mRNA expression levels and drug response but not significant PC); TRs in the purple set additionally had *RePhine* significant multi*P* (within the *RePhine* multivariate EN cutoff multi*P* < 0.005), and thus were predicted to be involved in the drug response independently (named *RePhine*-correlated-only independent TRs) (Figure 2B, Figure S5D). Group 3 contained the PC-*RePhine*-shared-correlated TRs (Figure 2B, red set, common TR candidates).

We then performed protein–protein interaction (PPI) analysis to investigate the biological connections among the TR candidates within each set. *RePhine*-correlated-only independent TRs (purple set) had more significantly enriched PPIs than PC-correlated-only TRs (Table S3). Both the *RePhine*-correlated-only independent TRs (purple set) and the PC-*RePhine*-shared-correlated TRs (red set) had over-represented pairwise PPIs (Figure 2C, Figure S6A). The pairwise PPIs achieved higher enrichment by pooling such TRs together (*P* = 2.58E − 10, derived from STRING database; Figure 2C and D; Table S3), suggesting tighter biological connections. In contrast, there were less significantly enriched PPIs observed among the CA-correlation-only TRs (green set) and among the CA-correlation-only and PC-*RePhine*-shared-correlated TRs (green and red sets) (Figure S6B and C; Table S3), suggesting unrelated and random connections between them. In addition, FOS and STAT3, two known biomarkers of erlotinib [16], [24], [25], [26], had tight interactions with the other candidates (Figure 2C and D).

### *RePhine* has an improved performance in CCLE dataset

To comprehensively evaluate the *RePhine* performance in real data, we next conducted PPI enrichment analyses to the TR candidates from PC analysis, GSEA, and *RePhine* for all drugs in CCLE dataset (Table S4).

*RePhine*-correlated independent TRs (having both significant uni*P* and significant multi*P*; see Method for details) displayed a significantly higher PPI enrichment than PC-correlated TRs (*P* = 0.003575, derived from STRING database; Table S4). However, this enrichment was not significantly higher than that of GSEA-correlated TRs. The results are reasonable because GSEA candidates contain redundant TRs such as subunits of the same complex, which tend to have more PPIs but *RePhine* only identifies the independent TRs among these redundant TRs. To test this notion, we compared the *RePhine*-correlated TRs that only had significant uni*P* (where functionally redundant TRs were not removed) with GSEA-correlated TRs. The results showed that the *RePhine*-correlated TRs had a significantly higher PPI enrichment than GSEA-correlated TRs (*P* = 6.52E − 05, derived from STRING database; Table S4). The significantly higher PPI enrichment of *RePhine*-correlated TRs suggests that TRs identified by *RePhine* have tighter biological connections and functional consistency.

To further evaluate the effectiveness of *RePhine*, we next performed PPI enrichment analyses (Table S4) and literature searching for CCLE drugs with marketing approval (Table S5) to compare *RePhine* with a published effective method “DoRothEA” [27]. Both methods were applied to CCLE data. *RePhine*-correlated independent TRs had a significantly higher PPI enrichment (*P* = 0.01488, paired *t*-test; Table S4), and the count of publication-consistent TRs (the true positives) of *RePhine* was higher than that of DoRothEA (38 hits *vs.* 12 hits; *P* = 1.078E − 04, Fisher’s test). In contrast, the counts of the false negatives and the candidates without literature support of *RePhine* were lower (16 hits *vs.* 36 hits, *P* = 0.004141, Fisher’s test; 55 hits *vs.* 80 hits, *P* = 0.00942, Fisher’s test; Table S5). The counts of the contrary predictions of both methods (where the predicted resistance or sensitivity were contrary to the publications) were similar (7 hits *vs.* 13 hits).

### *RePhine* effectively clusters drugs with similar action mechanisms

Next, we took advantage of *RePhine* to explore the relationship between TR regulation and drug response. We defined the TR significance profiles of *RePhine* correlation (−Log_10_ uni*P* with direction) across all CCLE drugs as TR response signatures. The response signatures reflect whether each drug has a specific TR response pattern. We computed the response signatures for all ENCODE TRs (*n* = 160). Notably, we did not filter TRs in this procedure. *RePhine* uni*P* values were calculated for all 160 TRs.

**Figure 3:**
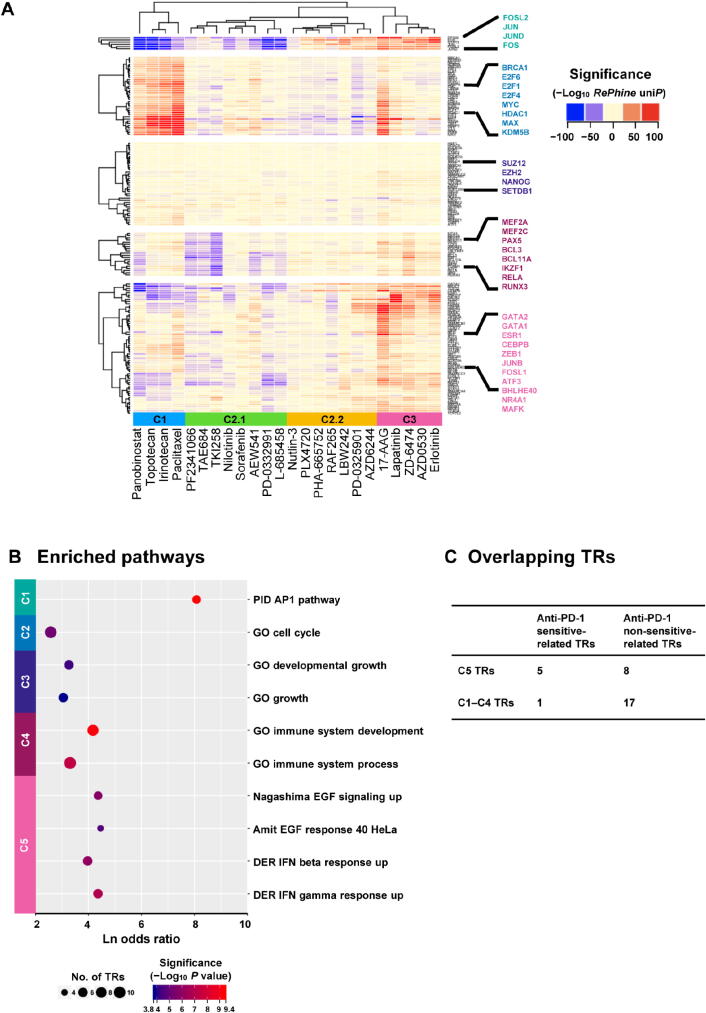
***RePhine-*characterized TR response signatures of drug clusters and functional enrichment. A.** Heatmap of *RePhine*-characterized response signatures of all TRs across the drugs. Blue and red colors indicate negative and positive *RePhine* correlations, respectively. Some well-known TRs in each cluster are highlighted. “ward.D” and “complete” methods were used to cluster the TRs and drugs, respectively. **B.** Enriched pathways of TRs in each cluster. *P* value was obtained from Fisher’s test. **C.** Summary of overlapping TRs between clusters 1–5 TRs and anti-PD-1 response-related TRs.

Our results suggest that drugs with similar action mechanisms tend to have similar TR signatures and *RePhine* can effectively cluster drugs with similar action mechanisms together. *RePhine*-based clustering separated the drugs into three clusters (Figure 3**A**). In cluster 1, all chemotherapy drugs as well as an HDAC inhibitor (panobinostat) were clustered together but away from other targeted therapy drugs. In cluster 3, drugs targeting EGFR (erlotinib, AZD0530, lapatinib, and ZD-6474) [28], [29], [30] were clustered together with an HSP90 inhibitor (17-AAG). In cluster 2, there were two subgroups. Cluster 2.1 contained two ALK inhibitors (PF-2341066 and TAE684), an ABL inhibitor (nilotinib), a CD4/6 inhibitor (PD-0332991), an IGF-1R inhibitor (AEW541), and two multi-kinase inhibitors (sorafenib and TKI258) [28]; in cluster 2.2, two MEK inhibitors (PD-0325901 and AZD6244), two RAF inhibitors (PLX4720 and RAF265), an MET inhibitor (PHA-665752), which were related to MAPK signaling [31], were clustered. All the drugs in cluster 2 were targeted therapy drugs [28]. In addition, clustering based on *RePhine* signatures had an improved performance compared with GSEA-based clustering in the mechanism-focused separation of the CCLE drugs (Figure 3A, Figure S7; File S1).

Hierarchical clustering analysis further separated 160 TRs into five clusters, which were associated with different types of therapies (Table 1). Each cluster showed enriched pairwise PPIs and functional consistency (Figure 3B; Table S6). For example, TR response signatures in chemotherapy drugs were associated with cell cycle as expected (Figure 3A and B), indicating that the cell cycle-related TRs could significantly regulate chemotherapy response [32]; the TRs in cluster 1 enriched in AP1 pathway were positively correlated with the response to both BRAF inhibitor and EGFR inhibitors. The TRs in cluster 5 that are related to EGF response and IFN-related pathways were only associated with the response to EGFR inhibitors (Figure 3A and B).

**Table 1:** Hierarchical clustering of TRs associated with different types of therapies.

**Cluster**	**Characteristic of TRs**
1	TRs with *RePhine* correlation to most MAPK inhibitors such as PLX4720 and erlotinib
2	TRs with positive *RePhine* correlation to chemotherapy drugs and cell cycle inhibitors
3	TRs with comparatively moderate *RePhine* correlation to drugs
4	TRs with positive *RePhine* correlation to EGFR inhibitors but with negative correlation to ABL and ALK inhibitors
5	TRs with positive *RePhine* correlation to EGFR inhibitors and MEK inhibitors but with negative correlation to chemotherapy drugs and CDK inhibitors

*Note*: TR, transcriptional regulator; MAPK, mitogen-activated protein kinase; EGFR, epidermal growth factor receptor; ABL, Abelson tyrosine kinase; ALK, anaplastic lymphoma kinase; MEK, mitogen-activated protein kinase kinase; CDK, cyclin-dependent kinase.

How can this TR clustering inform regarding response to anti-PD-1 immunotherapy? To answer this question, we integrated *in vivo* anti-PD-1 CRISPR screening data and patient-level anti-PD-1 response data from two previous studies [33], [34]. By comparing these TR clusters with CRISPR screening candidates, we found that the top 5 TRs (STAT1, SMARCA4, STAT2, GTF2F1, SMARCC1) whose loss-of-function would trigger the resistance to anti-PD-1 therapy were enriched in cluster 5 (*P* = 0.034, one-tail Fisher’s test; Figure 3C). To validate this observation, we explored the associations of these TRs with anti-PD-1 drug response in the patient cohorts [33]. Although these TRs were not observed to be differentially expressed between responders and non-responders, mutations of the genes encoding TRs in cluster 5 exclusively resulted in lower probability of complete response to anti-PD-1 therapy than that of partial response and progressive disease, which was identified through a multivariate logistical analysis by accounting for mutation loading (*P* = 0.0144, Table S7).

These results suggest that TRs particularly associated with response to EGFR inhibitors (Table 1, cluster 5) are also linked with anti-PD-1 effect [34]. Interestingly, it has been reported that EGFR pathways can positively regulate the activation of PD-1/PD-L1 pathway [35], [36]; such studies may explain why there is a common TR response signature between EGFR inhibitors and anti-PD-1.

### Identification of EZH2 as a BRAF inhibitor resistance-related TR

**Figure 4:**
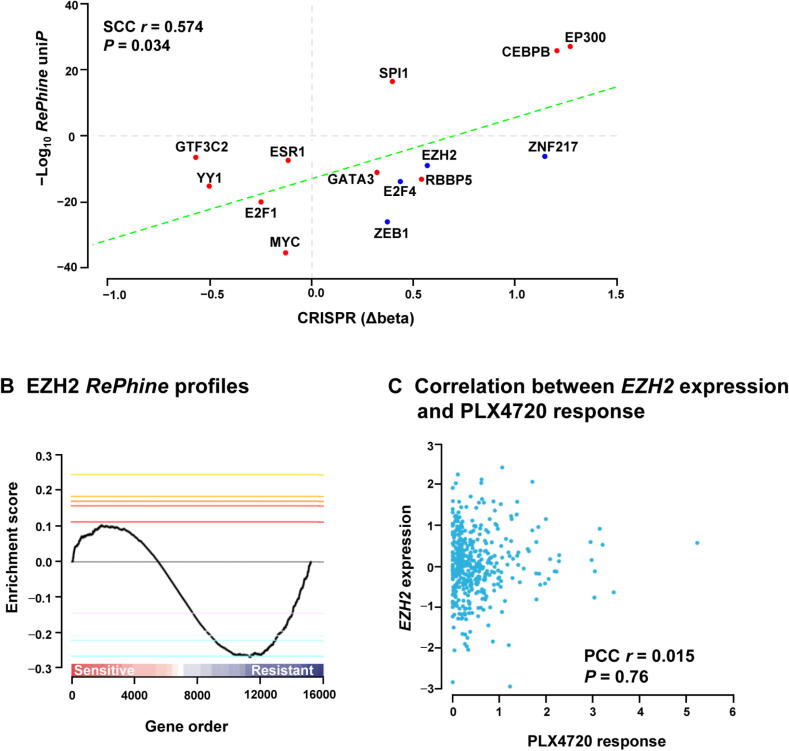
**EZH2 is predicted as a BRAF inhibitor resistance-related TR by *RePhine.* A.** Scatter plots showing the *RePhine* predicted response to PLX4720 of the top candidates (uni*P* < 1E − 5, multi*P* < 0.005, permu*P* < 0.05) and the TR knockout effects on PLX4032 response derived from the CRISPR screening data. The X-axis represents the difference of beta scores between drug treatment and control. Higher scores represent increased degrees of drug resistance affected by the gene knockout. Y-axis represents the − Log_10_*RePhine* uni*P* with direction (sensitive or resistant) of TRs related to drug response from *RePhine*. **B.** Visualization of EZH2 enrichment pattern. All genes were ordered by partial correlations from positive to negative. Maximum deviation from zero is defined as enrichment score. Targets of EZH2 have concordant negative partial correlations. **C.** Scatter plot showing correlation between *EZH2* expression and PLX4720 response. permu*P*, permutation *P* value.

PLX4720, the precursor of vemurafenib (PLX4032), is an ATP-competitive BRAF inhibitor. Resistance to BRAF inhibitors may rapidly develop in patients, but the mechanisms underlying this resistance are not fully understood [37], [38], [39]. We applied *RePhine* to PLX4720 using CCLE data and ENCODE ChIP-seq data, and identified GTF3C2, YY1, ESR1, E2F1, MYC, GATA3, RBBP5, EZH2, E2F4, ZEB1, and ZNF217 as the *RePhine*-negatively-correlated independent TRs (*RePhine* coefficient < 0, uni*P* < 1E − 5, multi*P* < 0.005, Figure 4**A**; Table S8). Among them, EZH2, E2F4, ZEB1, and ZNF217 are primarily transcription repressors (Table S9). On the other hand, activations of SPI1, CEBPB, and EP300 were determined as PLX4720 sensitivity predictors (*RePhine* coefficient > 0).

To validate all these candidates obtained from *RePhine*, we integrated the CRISPR-meditated gene knockout screening results for these candidates in A375 cells with PLX4032 treatment [37]. We used the MAGeCK method to interpret the CRISPR results [40]. Beta scores from MAGeCK indicate sgRNA abundances in the screen and the differences of beta scores between treatment and control reflect the effects of the drug on cell survival after gene knockout [40]. *RePhine*-identified candidates including resistant-related TRs as well as sensitive-related TRs were all observed to correspond well with the differences of beta scores from CRISPR results (Figure 4A). These results indicate that *RePhine* can effectively identify drug response-related TRs.

### Validation of EZH2 as a PLX4720 resistance-related TR by drug experiments

Our *RePhine* results showed that EZH2 is related to PLX4720 resistance. We observed that EZH2 had negative *RePhine* correlation with the PLX4720 response (Figure 4B); however, its mRNA levels did not exhibit any correlation with the PLX4720 response (Figure 4C). Therefore, we performed experimental validation of this *RePhine* prediction.

**Figure 5:**
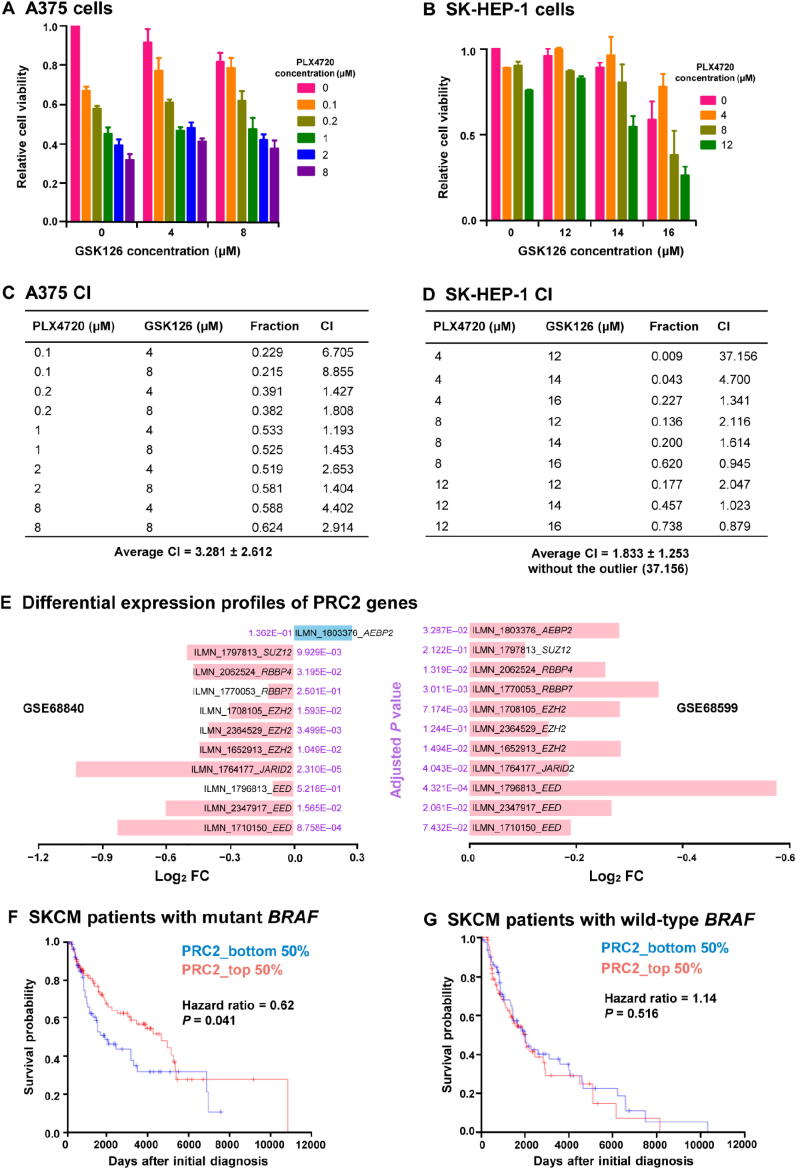
**Validation of EZH2 as a BRAF inhibitor resistance-related TR. A.** and **B.** Antagonistic inhibitory effect of the BRAF inhibitor PLX4720 and the EZH2 inhibitor GSK126 in A375 cells (A) and SK-HEP-1 cells (B). Cell viability was normalized to untreated cells at 48 h. **C.** and **D.** CI and fraction of A375 cells (C) and SK-HEP-1 cells (D) affected by drugs at different concentration combinations. Average CI (mean ± SD) was shown below. CI > 1 indicates antagonism. **E.** Down-regulation of PRC2 gene in BRAF inhibitor-resistant cells. **F.** and **G.** Kaplan-Meier plots for SKCM patients with mutant *BRAF* (F) and wild-type *BRAF* (G). Lower PRC2 activity scores were associated with worse outcomes in *BRAF* mutant group but not in the *BRAF* wild-type group. CI, combination index; FC, fold change; SKCM, skin cutaneous melanoma.

To experimentally validate the *RePhine* prediction of EZH2, we performed drug combination experiments with GSK126 and PLX4720. GSK126 is a potent and highly selective EZH2 inhibitor [41]. In both *BRAF* V600E mutant cell lines, A375 and SK-HEP-1, treatment with PLX4720 and GSK126 simultaneously resulted in antagonistic inhibitory effects at most dosage combinations (average CI = 3.281 for A375; average CI = 1.833; Figure 5**A–D**). In contrast, in the *BRAF* wild-type cell line JHH-7, there was no strong antagonistic interaction at most dosage combinations (average CI = 1.021), which suggests that the antagonistic effect is BRAF activation-dependent (Figure S8A and B). Contrary to our expectations, PLX4720 and GSK126 showed slight synergistic effect at high GSK126 dosages in SK-HEP-1 and JHH-7 (control) cell lines. This is possibly due to EZH2 being functional at the high dosage or existence of off-target pathway inhibition [42]. Similar antagonistic interactions were observed in two previous studies: experiments in cell lines [43] and *in vivo* mouse models [44]. It was worth pointing out that such studies claimed synergistic effect between BRAF inhibitors and EZH2 inhibitors in a subset of cancers that had *EZH2* amplification or gain-of-function mutations which led to re-distribution of H3K27me3 [44]. Their control experiments (in cell lines with wild-type *EZH2*) supported our finding, but the studies’ authors did not comment on their control findings.

### Validation of EZH2 as a PLX4720 resistance-related TR by bioinformatics analyses

We next validated the EZH2 role in PLX4720 resistance by analyzing the EZH2 ChIP-seq targets selected by *RePhine*, the differentially expressed genes in PLX4720-resistant cell lines, the relationship between PLX4720 response and PRC2 gene mutations in the CCLE data, and the effect of PRC2 deficiency on clinical outcomes in patients.

Given that EZH2 is a methyltransferase for H3K27 [42], we analyzed the EZH2 ChIP-seq targets selected by *RePhine* (Table S10). Besides the H3K27me3 signatures, the EZH2 ChIP-seq targets were enriched in several cancer-related pathways (Figure S8C; Table S10). Interestingly, knockout of genes encoding PRC2 essential subunits and other H3K27me3-related TRs, including ZNF217 [45], [46], also drove the cells to become comparatively resistant to BRAF inhibitor treatment according to the CRISPR-mediated gene knockout data (Figure S8D; Table S11).

We also discovered that *EZH2* and genes encoding other PRC2 subunits were significantly down-regulated in the BRAF inhibitor-resistant cells in two independent expression datasets GSE68840 and GSE68599 (Figure 5E).

In the analysis of PLX4720 response and PRC2 gene mutations in the CCLE data, we found that cell lines containing mutations in both *BRAF* and H3K27me3-related genes had significantly lower PLX4720 response than those with only *BRAF* mutations (*P* = 0.044, *t*-test; Figure S8E).

In TCGA skin cutaneous melanoma (SKCM) patient cohort dataset, where around half of the patients gained *BRAF* mutations, we investigated whether PRC2 deficiency affects clinical outcomes in patients. We defined an activity score for each patient to evaluate the activity of PRC2. Patients with relatively lower PRC2 activity scores had worse outcomes than those having higher scores in the *BRAF* mutant group (hazard ratio = 0.62, *P* = 0.041, 95% confidence interval: 0.40–0.98; Figure 5F). In contrast, the PRC2 activity scores were not predictive of overall survivals in the *BRAF* wild-type group (hazard ratio = 1.14, *P* = 0.516, 95% confidence interval: 0.76–1.71; Figure 5G), consistent with the results of the univariate Cox regression analysis (Figure S8F).

## Discussion

Due to low mRNA abundance of TRs and complexity of biological regulation mechanisms, detecting the linkage between TRs and drug response is still challenging. In this study, *RePhine* was developed to effectively perform three main tasks: 1) an integrative analysis on ChIP-seq targets to produce TR identification robust toward noise and complicated protein modifications, 2) an accurate measurement of correlation patterns by adjusting all potential CFs that are not under the impact of TR regulation, and 3) application of quantitative and informative target inference by considering both ChIP-seq signals and the distances from peaks to the targets to achieve a better evaluation of the associations.

There are still areas for further improvement. First, although significant correlations between TRs and pharmacological profiles could be detected through *RePhine* by exploring targets’ profiles, the relationship may not be causal. Some upstream regulators or kinases may exist that influence the drug response and simultaneously regulate downstream TRs. Therefore, TR target analyses and TR knockout or activation experiments combined with drug response examination are also required to validate the causal relationship. Second, because *RePhine* identifies drug response-related TRs through the targets, it is restricted to the cases where reliable target information is available. Lack of ChIP-seq data or ChIP-seq data with too few targets would mislead the identification of TRs. Hence, some existing target prediction algorithms could be exploited and complemented to facilitate target inference. Third, detection of acquired resistance is limited due to lack of post-treatment data in CCLE [28]. It is not trivial to detect the secondary alterations in response to drug treatment, which may elucidate why secondary resistance to erlotinib through a{Azuaje, 2017 #474}cquired STAT3 activation [47] could not be detected by *RePhine*. In addition, due to lack of post-treatment data, it is hard to integrate the effect of drug perturbations on genes [28], [48]. However, the pre-treatment correlation identified by *RePhine* may still be relevant to such drug influence on TRs. For example, FOS is selectively up-regulated by EGF stimulation and inhibited by EGFR TKI treatment in sensitive cells rather than in resistant cells [16]. If FOS could not be inhibited by EGFR TKI, the cells with higher FOS levels would not be sensitive to EGFR inhibition, and there would be no correlation between FOS activity and drug response. Nevertheless, such associations need further evaluation when post-treatment data are available.

*RePhine* has been further applied to an independent unpublished liver cancer dataset containing RNA-seq data, copy number information, and DNA sequencing data from more than 50 primary liver cancer cells coupled with pharmacological profiles for nearly 100 anti-cancer drugs [49]. Our novel identification, which has been validated by experiments, is that MYC promotion could independently and significantly increase the response of three drugs: BI-2536, PF-562271, and panobinostat. However, *MYC* mRNA did not show any correlations with the pharmacological profiles (Figure S9). The positive results obtained by applying *RePhine* to this liver cancer dataset further suggest that *RePhine* is an effective method for identifying drug response-related TRs and could be used in other independent pharmacogenomic data.

Code availability.

*RePhine*, which is implemented as an R package and accompanied by a user guide, is available at https://github.com/coexps/RePhine. RP scores, TCGA differentially expressed gene sets, modified Python scripts of RP score calculation, and R code for simulation and application for CCLE data are also available at https://github.com/coexps/RePhine.

Competing interests.

The authors have declared no competing interests.

### CRediT authorship contribution statement

**Xujun Wang:** Conceptualization, Methodology, Formal analysis, Software, Writing – original draft, Writing – review & editing. **Zhengtao Zhang:** Validation, Investigation. **Wenyi Qin:** Writing – original draft, Writing – review & editing. **Shiyi Liu:** Software. **Cong Liu:** Methodology. **Georgi Z. Genchev:** Conceptualization, Methodology, Formal analysis, Writing – original draft, Writing – review & editing. **Lijian Hui:** Validation, Investigation. **Hongyu Zhao:** Conceptualization, Methodology, Writing – original draft, Writing – review & editing, Supervision. **Hui Lu:** Conceptualization, Methodology, Formal analysis, Writing – original draft, Writing – review & editing, Supervision.

## Supplementary material

Supplementary File S1 **Supplementary information and methods**.

Supplementary data 2 **Expression patterns of the known TRs related to erlotinib response**. A. and B. Scatter plots showing the correlations between erlotinib response and mRNA levels of two transcriptional regulators (**A**) ZEB1 and (**B**) FOS.

Supplementary Figure S2 **Graphical representation of the RePhine workflow**. The detailed graph showing the input data, output data, and all the steps. The pink rounded rectangles represent the raw input data. The blue rounded rectangles represent the intermediate output. The black rounded rectangle represents the final output of transcriptional regulators (TRs). The green rectangles represent the steps of the workflow corresponding to the method sections. The grey arrows represent the input and purple arrows represent the output.

Supplementary Figure S3 **Distinguishing patterns of TRs by examining whether TR targets have concordant partial correlations**. A. Illustration of transcriptional regulator (TR) patterns. The targets are ordered by partial correlation coefficients. The accumulation of D_hit_ - D_miss_ was then calculated (the Y-axis, details in methods). The enrichment score is determined by the maximum deviation from zero. Lines with warm colors or cool colors represent permutation distributions of the negative and positive enrichment scores receptively. B.–D. Three typical TR patterns with enrichment that is: negative (B), positive (C), and none (D).

Supplementary Figure S4 **RePhine performance evaluation in simulated data**. RePhine has an improved performance in comparison with correlation analysis, logistic model, and GSEA. The RePhine performance in the simulated data. Blue, green, yellow and brown bars represent correlation method “Corr”, logistic methods “Logistic”, gene set enrichment analysis (GSEA) and RePhine method respectively. The asterisk represents significance levels (*, P < 0.05; **, P < 0.01; ***, P < 0.001). In the scenario (A and B, F–H), the counts of the simulated positive (drug response-related) and negative (drug response-non-related) TRs are both 100. In the scenario (I) the counts of the simulated positive and negative TRs are 10 and 100 respectively. A. AUC of the ROC of all the methods. RePhine shows an improved performance in the scenario with the confounders (P = 9.083E−05, 3.389E−05, 0.02868 as RePhine vs. correlation analysis (CA), logistic and GSEA respectively; one-side paired t-test is used as follows). The assumed correlation coefficient is fixed to 0.15, low level of expression noise is added (level = 2) to synergize with the effect of the confounders. No noise is added to regulatory potential (RP scores). The target count is 500 (5% of all 10000 genes). B. RePhine has an improved performance than CA and logistic regression model with the noise of the expression (P = 0.002189 and 0.001749 as RePhine vs. CA and logistic respectively. The correlation coefficient is set to 0.3 (before the noise exposure), No noise is added to RP scores and the target count is 500. C. ROC of RePhine and CA with different correlation coefficients. Levels of the noises added to expression and RP scores (sampling from real data) are both 5 in this scenario. The target count is 500. D. the distribution of the noise added to RP scores derived from the standard deviations of the replicates of the ChIP-seq data. E and F. RePhine has the better performance than GSEA with the noise of RP scores following either of the distribution (normal distribution: P = 0.009114; sampled real data P = 0.02308). The correlation is set to 0.3 and the expression noise is not added. The target count is 500. G. illustration of the RePhine performance under conditions with different numbers of TR targets. Levels of the noises added to expression and RP scores are both set to 5. Correlation is set to 0.3. H. AUC of the ROC of all the methods with multiple noises in balanced data (P = 0.002716, 0.00353, 0.009918 as RePhine vs. Corr, logistic and GSEA respectively). I. AUC of the precision-recall curve of all the methods with multiple noises in imbalanced data (P = 0.009075, 0.006167, 0.0181 as RePhine vs. Corr, logistic and GSEA respectively). Correlation is set to 0.3 in both balanced dataset and imbalanced dataset.

Supplementary Figure S5 **Comparison of RePhine uniP and correlation coefficients between TR mRNA levels and drug response in CCLE dataset**. A. Distributions of Spearman correlation coefficients between RePhine univariate P value (uniP, in log scale with the direction of sensitivity or resistance) and Pearson correlation coefficient between transcriptional regulator (TR) mRNA level and drug response in all 24 Cancer Cell Line Encyclopedia (CCLE) drugs illustrated by the violin plot. Each dot stands for the correlation coefficient between univariate P value (uniP) and coefficients of CA across all the TRs in the given drug. (B) and (C) are two example drugs among the 24 drugs and illustrated as the scatter plots for the significance (uniP) of TR-RePhine correlation (X-axis) and correlation coefficients of CA (Y-axis) respectively. B. Patterns of erlotinib. Each dot is a TR. C. Patterns of paclitaxel. D. top candidates with multivariate p-value (multiP) from RePhine results. Purple TRs are additionally within RePhine multivariate significance cutoffs (mulP < 0.005) but are not as significant in correlation analysis (CA). TRs that were not selected by the Elastic-Net model were in grey. The X-axis and Y-axis are the multiP and correlation P value (corP) on −Log10 scales respectively.

Supplementary Figure S6 **PPI networks among candidate TRs from RePhine and correlation analysis**. Panels A and B illustrated protein-protein interaction (PPI) networks among (A) CA-RePhine-shared-correlated transcriptional regulators (red TRs in Figure 2c) and (B) CA-correlated-only TRs (green TRs). C. PPI network by pooling the green and red TRs together.

Supplementary Figure S7 **Heatmap of all TR response signatures derived from GSEA enrichment scores (ES)**. Blue colors represent negative values of enrichment scores (ES) for transcriptional regulators (TRs) in the drug from gene set enrichment analysis (GSEA). Red colors represent positive values of ES. Color scale represents the degree of enrichment scores. Clusters are shown under the heatmap.

Supplementary Figure S8 **EZH2 and H3K27me3-related genes are associated with BRAF inhibitor response**. A. Bar plot for different concentration combinations of PLX4720 and GSK126 in a BRAF wild-type cell line JHH-7. Cell viability was normalized to untreated cells at 48h. B. CI for drug combinations at different dosages for JHH-7 cell line. Average CI (mean ± SD) was shown below. CI = 1 indicates additive effect; CI < 1 indicates synergistic effect; CI > 1 indicates antagonism. C. The over-represented KEGG pathways and H3K27me3 signatures of top 2000 EZH2 targets. D. Visualization of ZNF217 pattern. ZNF217 has a negative RePhine correlation to PLX4720. E. Distributions of PLX4720 response in three groups. Group 1, cell lines with mutations in both BRAF and H3K27me3-related genes; Group 2: cell lines only with mutated BRAF; Group 3: cell lines with both wild-type BRAF and H3K27me3-related genes. F. Bar plot for hazard ratios determined by the Cox regression model. Error bars represent 95% confidence interval. SKCM patients with higher PRC2 activity scores have lower hazard of death in BRAF mutant group (hazard ratio = 0.70, 95% confidence interval: 0.49–0.98, P = 0.0374). In contrast, in BRAF wild-type group, PRC2 activity scores were not significantly associated with hazard of death (hazard ratio = 0.82, 95% confidence interval: 0.63–1.05, P = 0.117).

Supplementary Figure S9 **Application of RePhine in an independent liver cancer dataset**. A. Visualizations of RePhine-correlation of MYC in BI-2536, Panobinostat and PF-562271 respectively in liver cancer cell lines. B. Scatter plots showing the correlation between MYC expression and the drugs in order. There is no significant correlation exhibiting between MYC expression and drug response. C. Relative cell number under drugs treatment after MYC activation. The Y-axis is the relative cell number normalized to treatment with DMSO. Cell number decreasing after MYC activation indicates MYC sensitizes the cells to these drugs. GFP is the control without MYC activation.

## References

[1] Siegel R.L., Miller K.D., Jemal A. (2018). Cancer statistics, 2018. CA Cancer J Clin.

[2] Otto T., Sicinski P. (2017). Cell cycle proteins as promising targets in cancer therapy. Nat Rev Cancer.

[3] Gong Z., Ma Q., Wang X., Cai Q., Gong X., Genchev G.Z. (2018). A herpes simplex virus thymidine kinase-induced mouse model of hepatocellular carcinoma associated with up-regulated immune-inflammatory-related signals. Genes (Basel).

[4] Azuaje F. (2017). Computational models for predicting drug responses in cancer research. Brief Bioinform.

[5] Haibe-Kains B,El–Hachem N, Birkbak NJ, Jin AC, Beck AH, Aerts HJWL (2013). Inconsistency in large pharmacogenomic studies. Nature.

[6] Stransky N., Ghandi M., Kryukov G.V., Garraway L.A., Lehar J., Liu M. (2015). Pharmacogenomic agreement between two cancer cell line data sets. Nature.

[7] Tsankov A.M., Gu H., Akopian V., Ziller M.J., Donaghey J., Amit I. (2015). Transcription factor binding dynamics during human ES cell differentiation. Nature.

[8] Riquelme E., Behrens C., Lin H.Y., Simon G., Papadimitrakopoulou V., Izzo J. (2016). Modulation of EZH2 expression by MEK-ERK or PI3K-AKT signaling in lung cancer is dictated by different KRAS oncogene mutations. Cancer Res.

[9] Liu S., Wang X., Qin W., Genchev G.Z., Lu H. (2018). Transcription factors contribute to differential expression in cellular pathways in lung adenocarcinoma and lung aquamous cell carcinoma. Interdiscip Sci.

[10] Wang X., Yan Z., Fulciniti M., Li Y., Gkotzamanidou M., Amin S.B. (2014). Transcription factor-pathway coexpression analysis reveals cooperation between SP1 and ESR1 on dysregulating cell cycle arrest in non-hyperdiploid multiple myeloma. Leukemia.

[11] Cancer therapy resistance (2014). chasing epigenetics. Nat Med.

[12] Chang C.J., Hung M.C. (2012). The role of EZH2 in tumour progression. Br J Cancer.

[13] Yamaguchi H., Hung M.C. (2014). Regulation and role of EZH2 in cancer. Cancer Res Treat.

[14] Zhou Q., Liu M., Xia X., Gong T., Feng J., Liu W. (2017). A mouse tissue transcription factor atlas. Nat Commun.

[15] Morgillo F., Della Corte C.M., Fasano M., Ciardiello F. (2016). Mechanisms of resistance to EGFR-targeted drugs: lung cancer. ESMO Open.

[16] Jimeno A., Kulesza P., Kincaid E., Bouaroud N., Chan A., Forastiere A. (2006). *C-fos* assessment as a marker of anti-epidermal growth factor receptor effect. Cancer Res.

[17] Jiang P., Freedman M.L., Liu J.S., Liu X.S. (2015). Inference of transcriptional regulation in cancers. Proc Natl Acad Sci U S A.

[18] Liu C., Wang X., Genchev G.Z., Lu H. (2017). Multi-omics facilitated variable selection in Cox-regression model for cancer prognosis prediction. Methods.

[19] Zou H. (2006). The adaptive lasso and its oracle properties. J Am Stat Assoc.

[20] Tang Q., Chen Y., Meyer C., Geistlinger T., Lupien M., Wang Q. (2011). A comprehensive view of nuclear receptor cancer cistromes. Cancer Res.

[21] Zou H., Hastie T. (2005). Regularization and variable selection via the elastic net. J R Statist Soc B.

[22] Subramanian A., Tamayo P., Mootha V.K., Mukherjee S., Ebert B.L., Gillette M.A. (2005). Gene set enrichment analysis: a knowledge-based approach for interpreting genome-wide expression profiles. Proc Natl Acad Sci U S A.

[23] Foucquier J., Guedj M. (2015). Analysis of drug combinations: current methodological landscape. Pharmacol Res Perspect.

[24] Grandis J.R., Drenning S.D., Chakraborty A., Zhou M.Y., Zeng Q., Pitt A.S. (1998). Requirement of Stat3 but not Stat1 activation for epidermal growth factor receptor-mediated cell growth *in vitro*. J Clin Invest.

[25] Gao S.P., Mark K.G., Leslie K., Pao W., Motoi N., Gerald W.L. (2007). Mutations in the EGFR kinase domain mediate STAT3 activation via IL-6 production in human lung adenocarcinomas. J Clin Invest.

[26] Agulnik M., da Cunha S.G., Hedley D., Nicklee T., Dos Reis P.P., Ho J. (2007). Predictive and pharmacodynamic biomarker studies in tumor and skin tissue samples of patients with recurrent or metastatic squamous cell carcinoma of the head and neck treated with erlotinib. J Clin Oncol.

[27] Garcia-Alonso L., Iorio F., Matchan A., Fonseca N., Jaaks P., Peat G. (2018). Transcription factor activities enhance markers of drug sensitivity in cancer. Cancer Res.

[28] Barretina J., Caponigro G., Stransky N., Venkatesan K., Margolin A.A., Kim S. (2012). The Cancer Cell Line Encyclopedia enables predictive modelling of anticancer drug sensitivity. Nature.

[29] Formisano L., D'Amato V., Servetto A., Brillante S., Raimondo L., Di Mauro C. (2015). Src inhibitors act through different mechanisms in Non-Small Cell Lung Cancer models depending on EGFR and RAS mutational status. Oncotarget.

[30] Liu J., Wu J., Zhou L., Pan C., Zhou Y., Du W. (2015). ZD6474, a new treatment strategy for human osteosarcoma, and its potential synergistic effect with celecoxib. Oncotarget.

[31] Zhi J., Li Z., Lv J., Feng B., Yang D., Xue L. (2018). Effects of PHA-665752 and vemurafenib combination treatment on *in vitro* and murine xenograft growth of human colorectal cancer cells with BRAF^V600E^ mutations. Oncol Lett.

[32] Topham C., Tighe A., Ly P., Bennett A., Sloss O., Nelson L. (2015). MYC is a major determinant of mitotic cell fate. Cancer Cell.

[33] Hugo W., Zaretsky J.M., Sun L., Song C., Moreno B.H., Hu-Lieskovan S. (2017). Genomic and transcriptomic features of response to anti-PD-1 therapy in metastatic melanoma. Cell.

[34] Manguso R.T., Pope H.W., Zimmer M.D., Brown F.D., Yates K.B., Miller B.C. (2017). *In vivo* CRISPR screening identifies *Ptpn2* as a cancer immunotherapy target. Nature.

[35] Li X., Lian Z., Wang S., Xing L., Yu J. (2018). Interactions between EGFR and PD-1/PD-L1 pathway: implications for treatment of NSCLC. Cancer Lett.

[36] Akbay E.A., Koyama S., Carretero J., Altabef A., Tchaicha J.H., Christensen C.L. (2013). Activation of the PD-1 pathway contributes to immune escape in EGFR-driven lung tumors. Cancer Discov.

[37] Shalem O., Sanjana N.E., Hartenian E., Shi X., Scott D.A., Mikkelsen T.S. (2014). Genome-scale CRISPR-Cas9 knockout screening in human cells. Science.

[38] Johnson D.B., Menzies A.M., Zimmer L., Eroglu Z., Ye F., Zhao S. (2015). Acquired BRAF inhibitor resistance: a multicenter meta-analysis of the spectrum and frequencies, clinical behaviour, and phenotypic associations of resistance mechanisms. Eur J Cancer.

[39] Arozarena I., Wellbrock C. (2017). Overcoming resistance to BRAF inhibitors. Ann Transl Med.

[40] Li W., Xu H., Xiao T., Cong L., Love M.I., Zhang F. (2014). MAGeCK enables robust identification of essential genes from genome-scale CRISPR/Cas9 knockout screens. Genome Biol.

[41] G ö llner S, Oellerich T, Agrawal-Singh S, Schenk T, Klein HU, Rohde C (2017). Loss of the histone methyltransferase EZH2 induces resistance to multiple drugs in acute myeloid leukemia. Nat Med.

[42] Shen J.K., Cote G.M., Gao Y., Choy E., Mankin H.J., Hornicek F.J. (2016). Targeting EZH2-mediated methylation of H3K27 inhibits proliferation and migration of synovial sarcoma *in vitro*. Sci Rep.

[43] Yu H., Ma M., Yan J., Xu L., Yu J., Dai J. (2017). Identification of coexistence of *BRAF V600E* mutation and *EZH2* gain specifically in melanoma as a promising target for combination therapy. J Transl Med.

[44] Souroullas G.P., Jeck W.R., Parker J.S., Simon J.M., Liu J.Y., Paulk J. (2016). An oncogenic *Ezh2* mutation induces tumors through global redistribution of histone 3 lysine 27 trimethylation. Nat Med.

[45] Cohen P.A., Donini C.F., Nguyen N.T., Lincet H., Vendrell J.A. (2015). The dark side of ZNF217, a key regulator of tumorigenesis with powerful biomarker value. Oncotarget.

[46] Banck M.S., Li S., Nishio H., Wang C., Beutler A.S., Walsh M.J. (2009). The ZNF217 oncogene is a candidate organizer of repressive histone modifiers. Epigenetics.

[47] Lee H.J., Zhuang G., Cao Y., Du P., Kim H.J., Settleman J. (2014). Drug resistance via feedback activation of Stat3 in oncogene-addicted cancer cells. Cancer Cell.

[48] Iorio F., Knijnenburg T.A., Vis D.J., Bignell G.R., Menden M.P., Schubert M. (2016). A landscape of pharmacogenomic interactions in cancer. Cell.

[49] Qiu Z, Li H, Zhang Z, Zhu Z, He S, Wang X, et al. A pharmacogenomic landscape in human liver cancers. Cancer Cell 2019;36:179–93.e11. 10.1016/j.ccell.2019.07.001 PMC7505724 31378681

