# Supplementary material for: RePhine: An Integrative Method for Identification of Drug Response-related Transcriptional Regulators
Source: Genomics Proteomics Bioinformatics. 2021 Mar 10;19(4):534–48. doi: 10.1016/j.gpb.2019.09.008 (PMC9040019; doi:10.1016/j.gpb.2019.09.008)
Supplement: Supplementary File S1 — Supplementary information and methods. [file mmc1.docx]

**File S1 Supplementary information and methods**

**Supplementary information**

**RePhine has improved performance in comparison with some common methods in the simulation data**

To systematically examine whether RePhine can accurately identify the associations between the TRs and the drug response, the performance was evaluated in the simulation datasets and we compared RePhine with the some commonly used methods 1) correlation analysis (CA), 2) logistic regression model, and 3) Gene set enrichment analysis (GSEA) analysis. In the simulation setup, the expression of both the drug response-related TRs and their target genes were assumed to be both correlated with the drug response to make these methods comparable. Area Under Curve (AUC) of Receiver Operating Characteristic curve (ROC curve) results were compared. The RePhine metrics UniP and permuP were used to predict the significance of the associations. We evaluated the effect of some confounders, noise in expression and RP scores and the number of downstream targets on RePhine and the other methods. In addition, we also compared these methods in the scenario where multiple noise and confounders were added simultaneously.

*Confounders assessment*

Confounding mutations are known to affect drug response independently of TR regulation (such as kinase mutations) [1]. We first evaluated the robustness of all the methods with such confounders. *RePhine* has shown improved results in comparison to the other methods with the increase of confounder counts (Figure S4A). This result suggested confounders could significantly mislead the prediction of drug response-related TRs and should be accounted for.

*Robustness assessment*

To examine whether *RePhine* can provide robust identification of TRs, some noise was then added to the expression values of the genes and TRs or ChIP-seq RP scores.

Noise of gene expression

*RePhine* was compared to the other methods with the addition of noise to gene expression including TR targets, non-targets, and TRs. All the methods showed AUC more than 0.9 at a low level of expression noise (expression noise level = 1, Figure S4B). RePhine method significantly outperformed the CA (correlation analysis) and the logistic model with the increasing levels of the noise added to the expression (Figure S4B). Furthermore, we demonstrated that *RePhine* could effectively identify drug response-related TRs without the need for strong correlation patterns. For example, the *RePhine* method at a lower correlation of 0.3 could achieve a similar AUC from CA at a correlation of 0.5 (AUC *RePhine* 0.8435 *vs*. CA 0.8521, Figure S4C). These comparisons suggested that *RePhine* was more accurate and more noise-robust than CA and the logistic model. GSEA and *RePhine* had similar performance in this step because both methods are both target-inferred methods.

Noise of RP scores

Since *RePhine* and GSEA methods consider the target information, noise tolerance of target information in ChIP-seq between both methods was compared. Because the distribution of noise of RP scores had not been well-characterized, we used two strategies to simulate the noise of RP scores: one was that the noise followed the normal distribution; the other was that the noise was sampled from the real ChIP-seq data (Figure S4D, see the following methods for details). Although higher levels of noise of ChIP-seq could decrease the performance of both methods, *RePhine* demonstrated improved performance than GSEA in both distributions (Figure S4E and F).

*Number of RP targets effect*

We examined how the number of downstream targets with RP scores could affect performance. As expected, a larger number of targets contributed to higher prediction accuracy (Figure S4G).

*Multiple of noise and confounders added to balanced and imbalanced data*

To better evaluate the performance, we compared *RePhine* to the other methods with multiple noise and confounders added simultaneously. *RePhine* outperformed the other methods in both the balanced data (ratio of negative TRs *vs*. positive TRs = 100:100, Figure S4H) as well as in the imbalanced data (ratio of negative TRs *vs*. positive TRs = 10:100, Figure S4I).

These simulation results clearly demonstrated the advantage of *RePhine* over these commonly used methods. *RePhine* employs a novel strategy with careful consideration of noise and confounders and provides a robust identification of TRs.

***RePhine* derived TR response signatures are more correlated to the drug mechanisms in comparison with GSEA**

Clustering based on *RePhine* signatures had improved performance compared with GSEA based clustering in the mechanism-focused separation of the CCLE drugs. (Figure 3A, Figure S7). The results suggest that *RePhine* derived TR response signatures may be better correlated to the drug mechanisms. GSEA enrichment score-based clustering also resulted in separating the drugs into three groups. Similar to *RePhine*, GSEA clustered RAF inhibitors and MEK inhibitors together in cluster 3; clustered most EGFR inhibitors with the HSP90 inhibitor (17-AGG) in cluster 2; and clustered the chemotherapies with a HDAC inhibitor (panobinostat) in cluster 1.2 [2]. However, GSEA clustered the chemotherapies together with the multi-kinase inhibitor (sorafenib) and some other targeted drugs in cluster 1.2. Those treatment modalities have different mechanisms [3]. Besides, GSEA clustered another multi-kinase inhibitor (TKI258) with the ALK inhibitors (PF-2341066 and TAE684), and the ABL inhibitor (nilotinib) in cluster 1.1 [2].

**Supplementary methods**

**Genomic, clinical data, and pharmacology profiles**

We used CCLE datasets containing paired gene expression, copy number and mutation data of tumor cell lines, coupled with pharmacology profiles [2] (<https://portals.broadinstitute.org/ccle/home>). Expression and CNV data were normalized and centered to zero. Activity area values designated in the original paper were used in our analyses to measure the degrees of drug efficacy.

ChIP-seq data peak files were downloaded from the ENCODE database for the target inference of TRs [4]. ChIP-seq data in which more than 90% of the genes have the called peaks within 100kb to the transcription start sites were removed. TCGA SKCM data RNA-seq level 3 data and clinical data were obtained from the Firebrowse database (<http://firebrowse.org/)>. Immunotherapy data were obtained from supplementary data of original papers including the mutation information, the response information, and the CRISPR screening candidates [5,6].

Expression profiles of the BRAF inhibitor sensitive and resistant cell lines were obtained from Gene Expression Omnibus datasets. GSE68599 contains two BRAF inhibitor resistant cells and parental sensitive cells with two biologic replicates in each cell type. GSE68840 contains four biological replicates for resistant and three replicates for parental sensitive cells. Differentially expressed genes between sensitive and resistant cells were called by *limma* [7].

*RePhine* was implemented as an R package (Rephine). The *RePhine* R package accompanied with user guide is also available on GitHub. Sources used in the manuscript such as RP scores, TCGA differential genes, modified python scripts for RP scores calculation, and R code for simulation and application for CCLE data are available in GitHub (https://github.com/coexps/Rephine).

**RePhine workflow**

*Partial correlation calculation*

We calculated the partial correlation to measure the association between the adjusted expression (derived from Step 1.1) for a given gene and the response of a certain drug by controlling for the confounding variables selected in the adaptive lasso step (step 1.2.2). The detailed calculation is described as follows:

First, we solved two linear regression models.

$$\boldsymbol{w}_{X}^{*}=\arg\min_{\boldsymbol{w}} \left\{ \sum_{n=1}^{N} \left( x_{n}^{adj_{Expr}}-\left\langle\boldsymbol{w,}\boldsymbol{z}_{\boldsymbol{n}} \right\rangle\right)^{2} \right\};$$

$$\boldsymbol{w}_{Y}^{*}=\arg\min_{\boldsymbol{w}} \left\{ \sum_{n=1}^{N} \left( y_{n}^{drug}-\left\langle\boldsymbol{w,}\boldsymbol{z}_{\boldsymbol{n}} \right\rangle\right)^{2} \right\} (1)$$

where $x_{n}^{adj\_Expr}$ represents the adjusted expression for a given gene in cell line *n*; $y_{n}^{drug}$represents the response for a given drug in the cell line *n*; $\boldsymbol{z}_{\boldsymbol{n}}=\{z_{n}^{type}, z_{n}^{mutations}\}$ is the confounder vector; $z_{n}^{type}$is the dummy variable to distinguish the cancer types. 0 and 1 stand for leukemia and solid tumor respectively; $z_{n}^{mutations}$ is the mutation vector representing the mutation status of genes that were selected from the adaptive lasso model (Step 1.2.2).

Then the fitted residuals were extracted.

$$e_{X, n}=x_{n}^{adj\_Expr}-\left\langle\boldsymbol{w}_{X}^{*}\boldsymbol{,}\boldsymbol{z}_{\boldsymbol{n}} \right\rangle; e_{Y,n}=y_{n}^{drug}-\left\langle\boldsymbol{w}_{Y}^{*}\boldsymbol{,}\boldsymbol{z}_{\boldsymbol{n}} \right\rangle(2)$$

where $e_{X,n}$ and $e_{Y,n}$ represent the fitted residuals from their respective linear model.

Finally, the partial correlation coefficient across all the *N* cell lines was computed using the formula below:

$$\hat{\rho}_{XY\cdot Z=}\frac{N\sum_{n=1}^{N} e_{X,n}e_{Y,n}-\sum_{n=1}^{N} e_{X,n}\sum_{n=1}^{N} e_{Y,n}}{\sqrt{N\sum_{n=1}^{N} e_{X,n}^{2}-{(\sum_{n=1}^{N} e_{X,n})}^{2}}\sqrt{N\sum_{n=1}^{N} e_{Y,n}^{2}-{(\sum_{n=1}^{N} e_{Y,n})}^{2}}} (3)$$

R package “*ppcor*” was used to compute the partial correlation.

**Experimental cell lines and culture**

A375 is a melanoma cell line. SK-HEP-1 is a human cancer cell line of endothelial origin. JHH-7 is a human hepatocellular carcinoma cell line. A375 and SK-HEP-1 have BRAF V600E mutation, JHH-7 is BRAF wide type. All cell lines were regularly tested for being free of mycoplasma, and their identities were verified. A375 was purchased from Cell Bank of Chinese Academy of Sciences in Shanghai ([www.cellbank.org.cn](http://www.cellbank.org.cn), identifier SCSP-533). SK-HEP-1 was purchased from ATCC (<https://www.atcc.org>, Identifier HTB-52). JHH-7 was purchased from JCRB (<https://cellbank.nibiohn.go.jp/>, identifier JCRB1031). Both A375 and SK-HEP-1 were cultured in DMEM (Gibco) supplemented with 10% FBS (HyClone), 1.5g/L NaHCO3, 100U/ml penicillin and 100µg/ml streptomycin, JHH-7 was cultured in Williams' Medium E supplemented with 10% FBS, 100U/ml penicillin and 100µg/ml streptomycin. All cell lines were maintained in a humidified incubator at 37℃ with 5% CO2 and digested by 0.05% trypsin-EDTA for passage at the ratio of 1:3 every 3 days.

**Data simulation and analysis**

*Simulation and assessment*

Gene expression, random noise, target RP scores, expression of a drug response-related TR (positive), and a non-related TR (negative) were generated in each simulation run and independently repeated for 100 times. UniP, permuP were calculated by *RePhine*. Correlation p-value from correlation analysis between drug response and TR mRNA level (CA), logistic p-value, and GSEA permutation p-value were calculated to assess the significance of the TRs from each method. MultiP was not considered in the simulation step because each simulation was independent. The accuracy of the prediction was then evaluated by AUC. The maximum p-value of the *RePhine* uniP and permuP was assigned to TRs from the *RePhine* results. -log_10_ p-values of the methods were used during the rankings and AUC calculation. The R package *“rocr”* was used to evaluate the performance.

*Drug response*

The drug response values (1 x 300 vectors) were generated first, which follow normal distribution N (5,1). R function “rnorm” was used.

*TRs expression*

Expression of drug response-related TRs was simulated with a correlation coefficient r to the drug response values. In contrast, expression of drug response non-related TRs was not correlated to drug response values.

*TR targets and non-targets expression*

We simulated 10000 genes expression across 300 samples (10000 * 300 matrix). Among the genes, expression values of the targets belonging to drug response-related TRs were generated and set to be correlated to drug response values. *r_1_, r_2_, … r_n_* were the correlation coefficients of target_1_, target_2_ … target_n_, whose Fisher transformed values *V_1_, V_2_, … V_n_* followed N (*z’*, 1). z’= $\frac{1}{2}ln\left( \frac{1+r}{1-r} \right)$. *r* was the assumed correlation coefficient between target expression and drug response. Coefficients (after Fisher transformation) of non-related TR targets and all non-target genes were assumed to follow the standard normal distribution N (0,1):

$$r =\left\{ \begin{aligned} r_{n} \sim N \left( z’, 1 \right) if n \in target \\ r_{n} \sim N \left( 0, 1 \right) if n NOT \in target \end{aligned} \right.$$

where$z' =\frac{1}{2}ln\left( \frac{1+r}{1-r} \right)$.

*Regulatory potential scores*

The RP scores of TR targets were generated by R function “*runif*” which followed a uniform distribution U (0,10). The scores of genes didn’t belong to a TR (non-targets) were set to 0.

*Number of targets*

We adjusted the numbers of the targets during the simulation to examine the performance of *RePhine* on TRs with different target counts. For example, “5% targets” means 5% of 10,000 genes were regarded as the downstream targets of a simulated TR where such targets had higher RP scores.

*Noise simulation*

After the data were simulated, the noise of expression and RP scores were added to the data:

Noise levels of expression

Noise values were sampled from a normal distribution N (0, SD) and added to both genes and TR expression across the samples, where SD (standard deviation) represented the noise level.

Noise levels of ChIP-seq

We used two strategies and distributions to generate noise RP scores. 1) Denote ${Ns}^{RP}{=({Ns}_{1}^{RP},\ldots Ns}_{n}^{RP})$^T^ following normal distribution N (0, 1), and noise of RP scores = ${Ns}^{RP}* \delta$, where $\delta$ was the noise level. 2) Set ${Ns'}^{RP}{=({Ns^{'}}_{1}^{RP},\ldots Ns'}_{n}^{RP})$^T^ sampled from the standard deviation of ChIP-seq replicates, and noise of RP scores = ${Ns'}^{RP}* \delta'$ where $\delta'$ was the noise level. First, we selected the samples of the TRs with more than three replicates (of the same cell lines); second, we calculated the RP scores of each sample and standardized the values; third, we calculated the standard deviation (SD) of the replicates of each TR. Forth, we pooled all the SD together and sampled values from this pool.

*Confounders*

The confounders were simulated to mimic somatic mutations which might increase or decrease the drug response independent of the regulation by TRs. We assumed that 15% of the samples were affected by the confounders. We simulated the confounders whose count was 15% of the number of cell lines (e.g. 15% of 300). The values of the confounders followed the uniform distribution U(3,5) or U(-3,-5). Then the confounding values were added to the drug response values in random order. If the drug response value was less than zero after adding the confounders, we set such values to zero.

**Methods comparison description**

Both correlation analysis (CA) and logistic model were applied to calculate the correlation between TR mRNA and drug response. In the logistic analysis, cells were divided into 2 groups: sensitive cells (cells with top 50% response values) and resistant cells. The GSEA method was implemented from the original paper [8]. In the simulation data, genes with the highest *N* RP scores were considered as targets, where *N* was the assumed target count. In the real data comparison, all the genes with RP scores > 0 were regarded as the targets of this TR.

**RePhine enrichment score calculation and interpretation**

If $P_{j}^{reg}$ is binary 0 or 1, the formula of RePhine enrichment scores will reduce to GSEA. Here we supply the details as follows:

$$D_{miss}\left( TR,i \right)=\sum_{j\leq i} \left( 1-P_{j}^{reg} \right)\times\frac{1}{N-\sum_{j=1}^{N} P_{j}^{reg}}, where N is the gene counts$$

When $P_{j}^{reg}$ = ${(P}_{1}^{reg}, P_{2}^{reg}\ldots P_{i}^{reg})$^T^ is binary 0 or 1, the formula will reduce to GSEA: $P_{miss}\left( S,i \right)=\sum_{\begin{aligned} g_{j}\notin S \\ j\leq i \end{aligned}} \frac{1}{(N-N_{H})}$ .

If $P_{j}^{reg}$ = ${(P}_{1}^{reg}, P_{2}^{reg}\ldots P_{i}^{reg})$^T^ is $P_{j}^{reg}=1$and $\mathrm{gene}_{j}\in TR$, $D_{miss}\left( TR \right)\mathrm{in}\mathrm{gene}_{j}=0$.

If $\mathrm{gene}_{j}\notin TR$, $P_{j}^{reg}=0$ and $D_{miss}\left( TR \right)\mathrm{in}\mathrm{gene}_{j}=\frac{1}{N-\sum_{j=1}^{N} P_{j}^{reg}}= \frac{1}{N-N_{p=1}}$.

Therefore, $D_{miss}\left( TR,i \right)=\sum_{\begin{aligned} \mathrm{gene}_{j} \notin S \\ j\leq i \end{aligned}} \frac{1}{(N-N_{p=1})}$

**DoRothEA implementation and application to CCLE datasets**

TF activity of each CCLE cell line was calculated using the code in Github (<https://github.com/saezlab/DoRothEA>). Next, the TF effect on drug response was defined by the regression coefficient (β_TF_) estimated with a multiple linear least square’s regression [9]. X_covariates_ is the cancer type information. Y_drug_ is the drug response values of the cell line and ψ is the noise. P-values were adjusted for multiple testing corrections using the Benjamini-Hochberg method.

Ydrug = βcovariates * Xcovariates + βTFXTF + ψ

**GSEA analysis**

First, the correlation between gene expression and drug response was calculated. Correlation coefficients were used to rank the genes. Then, TR targets were regarded as the gene set of a given TR. Next, enrichment scores of GSEA were calculated to test whether such set of targets (gene set) showed statistically significant, concordant associations to drug response. The significance was obtained by the permutation test.

**PRC2 activity scores computation**

An activity score was defined to evaluate the extent of loss of PRC2 function by examining whether any of these subunits was relatively down-regulated. We first centered the SUZ12, EED, and EZH2 expression across the samples to zero. Then we determined the activity score of each patient by identifying the lowest expression of the three genes within one sample since any of the essential subunit deficiency could lead to functional loss.

$$score =min \left( {Suz12}_{j}-\frac{\sum_{j=1}^{n} {Suz12}_{j}}{n},{EED}_{j}-\frac{\sum_{j=1}^{n} {EED}_{j}}{n},{EZH2}_{j}-\frac{\sum_{j=1}^{n} {EZH2}_{j}}{n}, \right)$$

**Survival analysis**

We used the Cox model to explore the relationship between patient survival and PRC2 activity scores. Package “*survival*” in R was used to calculate the statistical significance. K-M plots were also generated through this package.

**Transcriptional repressors correction**

Since transcriptional repressors contribute to gene silencing, their activation results in the suppression of their target genes’ expression. When a given repressor is positively correlated with drug response, their targets expression level should be in opposite directions from the upstream repressor’s expression levels. To better evaluate the relationship between *RePhine* and Correlation analysis (CA), repressors were specially corrected by reversing the sign of the coefficient. A TR which primarily participates in gene silencing was regarded as a repressor. The repressors are listed in Table S9.

**Protein-protein Interaction analysis**

TRs from GSEA (ES > 0.2, *P* < 0.01), CA (*P* < 0.01) and DoRothEA (*P* < 0.01), RePhine (independent TRs: uniP < 1E–5, multiP < 0.005, permuP < 0.05, Non-independent TRs: uniP < 1E–5, permuP < 0.05) were evaluated using PPI enrichment to measure the biological connections among the candidates. If counts of the candidate TRs were more than 15, top 15 TRs would be used to make the counts comparable.

We queried the STRING database v10.5 (https://string-db.org/). PPI Enrichment *P* value among the candidates was directly generated from STRING webserver to evaluate whether a network has significantly enriched interactions than expected [10]. Visualization of protein networks was sourced from this database.

**Publication searching and criteria**

For literature-based comparison, we established a literature-supported TR-drug relationship list by searching for TR and drug treatment relationships that satisfy any of the following criteria 1) TR has been experimentally validated to be required to mediate sensitivity or resistance to the exact drugs or related drugs with the same mechanism of action; 2) TR has been documented to be up-regulated or down-regulated in resistant cells compared to sensitive cells in the main text; 3) Whether the drug has been reported to be effective or non-effective in TR-positive or TR-negative patient cohorts (*e.g.*, Estrogen-receptor-positive or Estrogen-receptor-negative).

Additionally, we excluded the following two scenarios from the literature-supported TR-drug relationship list. 1) when there was existence of conflicting reports of the function of the TR in drug treatment (*e.g.*, one paper reports a sensitizing effect and another an antagonistic effect); 2) when the expression of the TR is regulated by the drug treatment, but there is no report of a clear association between this TR and the drug (this criterion is to exclude cases in which the TR expression is affected by drug treatment but related to drug response). Only CCLE drugs with marketing approval and the predicted TRs from either method were compared because more research focuses on approved drugs.

**Results comparison among different parameters and procedures**

The consistency ratio "N_same_/N_all_TR_” was defined and used to measure the consistency of the results through different parameters and procedures. N_same_ represents the count of the consistent TRs in the comparison, N_all_TR_ was the counts of all TRs. To evaluate whether different $\alpha$ in Elastic-Net model influence the TR selection, we compared the results using $\alpha=0.2, 0.4, 0.6,\mathrm{and} 0.8$. In this comparison, both the Elastic-Net model directly selecting TRs and the significant TRs were compared. Accordingly, the results from the procedures with and without the filtering step 1.4 (pan-cancer genes filtering) were compared in all CCLE drugs.

**Experiments of the drug screening**

Before drug screening, the optimal number of seeding cells was determined to avoid over-confluency at the end of drug treatment (around 90% confluency). The cells were cultured in dishes and harvested for drug screening with 80%-90% confluency. Then the single-cell suspension was seeded in 384-well plates at the pre-determined cell density at the volume of 50µl by Multidrop Combi Reagent Dispenser (Thermo Fisher Scientific), after overnight incubation. Drug A with X doses and drug B with Y doses serial-diluted at fixed ratios from the top concentration were added into the 384-well plates by Bravo Automated Liquid-Handling Platform (Agilent), the plates were centrifuged at the speed of 800rpm for 2 min and transferred to the incubator for 72h drug treatment. At the endpoint of treatment, the cell viability was determined by CellTiter-Glo reagent (Promega), the 25µl mixed reagent was added to each well by Multidrop Combi Reagent Dispenser and after 10 min incubation in room temperature, the luminescent signals were detected by EnVision Multilabel Reader (PerkinElmer). The relative cell viability was presented as the ratio of the luminescent signal value of drug treatment to solvent control.

**Reference**

[1] Barnes TA, O'Kane GM, Vincent MD, Leighl NB. Third–Generation Tyrosine Kinase Inhibitors Targeting Epidermal Growth Factor Receptor Mutations in Non-Small Cell Lung Cancer. Front Oncol 2017;7:113.

[2] Barretina J, Caponigro G, Stransky N, Venkatesan K, Margolin AA, Kim S, et al. The Cancer Cell Line Encyclopedia enables predictive modelling of anticancer drug sensitivity. Nature 2012;483:603–7.

[3] Masui K, Gini B, Wykosky J, Zanca C, Mischel PS, Furnari FB, et al. A tale of two approaches: complementary mechanisms of cytotoxic and targeted therapy resistance may inform next-generation cancer treatments. Carcinogenesis 2013;34:725–38.

[4] Sloan CA, Chan ET, Davidson JM, Malladi VS, Strattan JS, Hitz BC, et al. ENCODE data at the ENCODE portal. Nucleic Acids Res 2016;44:D726–32.

[5] Manguso RT, Pope HW, Zimmer MD, Brown FD, Yates KB, Miller BC, et al. In vivo CRISPR screening identifies Ptpn2 as a cancer immunotherapy target. Nature 2017;547:413–8.

[6] Hugo W, Zaretsky JM, Sun L, Song C, Moreno BH, Hu-Lieskovan S, et al. Genomic and Transcriptomic Features of Response to Anti-PD-1 Therapy in Metastatic Melanoma. Cell 2017;168:542.

[7] Ritchie ME, Phipson B, Wu D, Hu Y, Law CW, Shi W, et al. limma powers differential expression analyses for RNA-sequencing and microarray studies. Nucleic Acids Res 2015;43:e47.

[8] Subramanian A, Tamayo P, Mootha VK, Mukherjee S, Ebert BL, Gillette MA, et al. Gene set enrichment analysis: a knowledge-based approach for interpreting genome-wide expression profiles. Proc Natl Acad Sci U S A 2005;102:15545–50.

[9] Garcia-Alonso L, Iorio F, Matchan A, Fonseca N, Jaaks P, Peat G, et al. Transcription Factor Activities Enhance Markers of Drug Sensitivity in Cancer. Cancer Res 2018;78:769–80.

[10] Szklarczyk D, Morris JH, Cook H, Kuhn M, Wyder S, Simonovic M, et al. The STRING database in 2017: quality-controlled protein-protein association networks, made broadly accessible. Nucleic Acids Res 2017;45:D362–D8.
