# Supplementary figures and images for: RePhine: An Integrative Method for Identification of Drug Response-related Transcriptional Regulators
Source: Genomics Proteomics Bioinformatics. 2021 Mar 10;19(4):534–48. doi: 10.1016/j.gpb.2019.09.008 (PMC9040019; doi:10.1016/j.gpb.2019.09.008)

**A**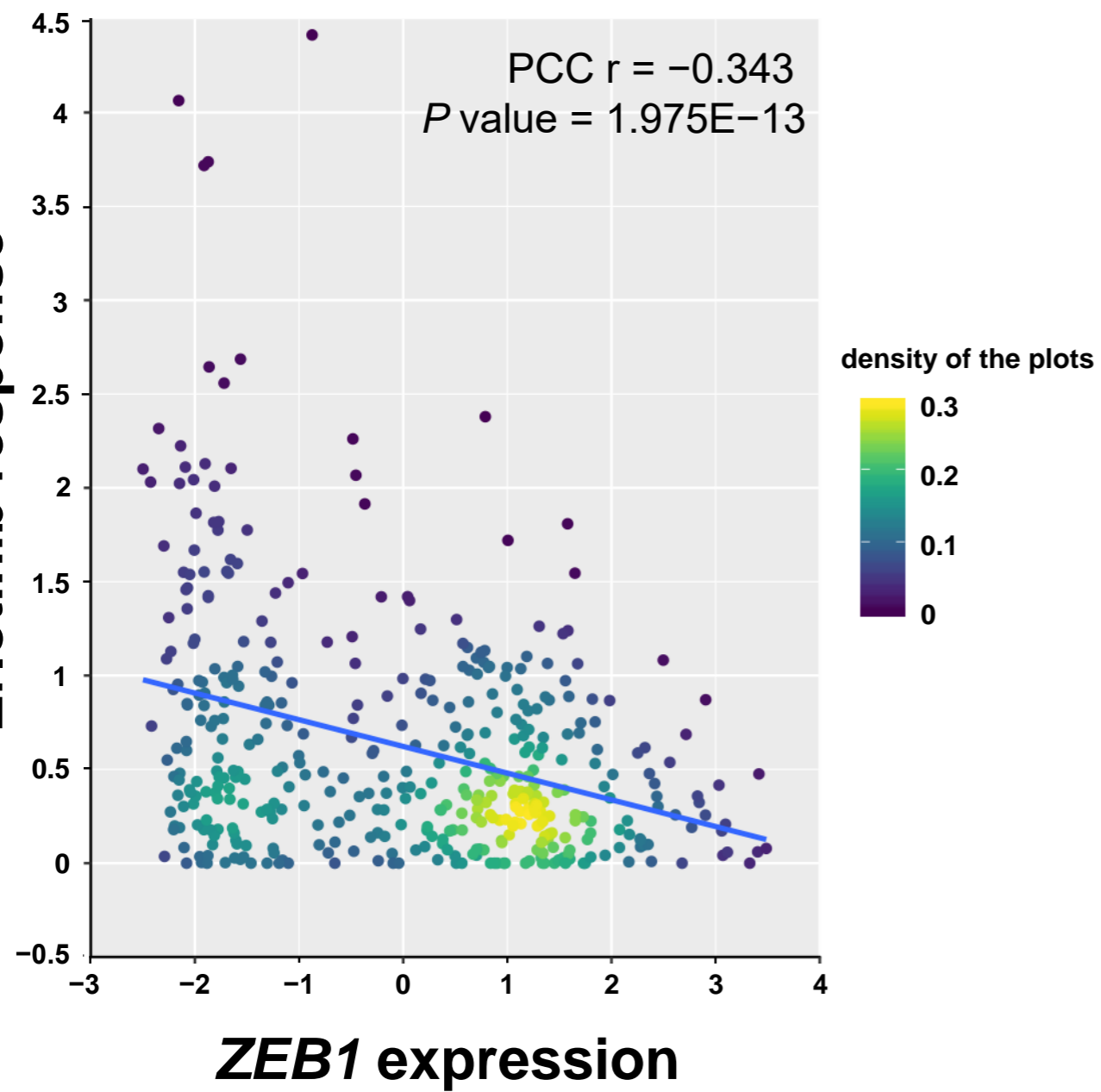**B**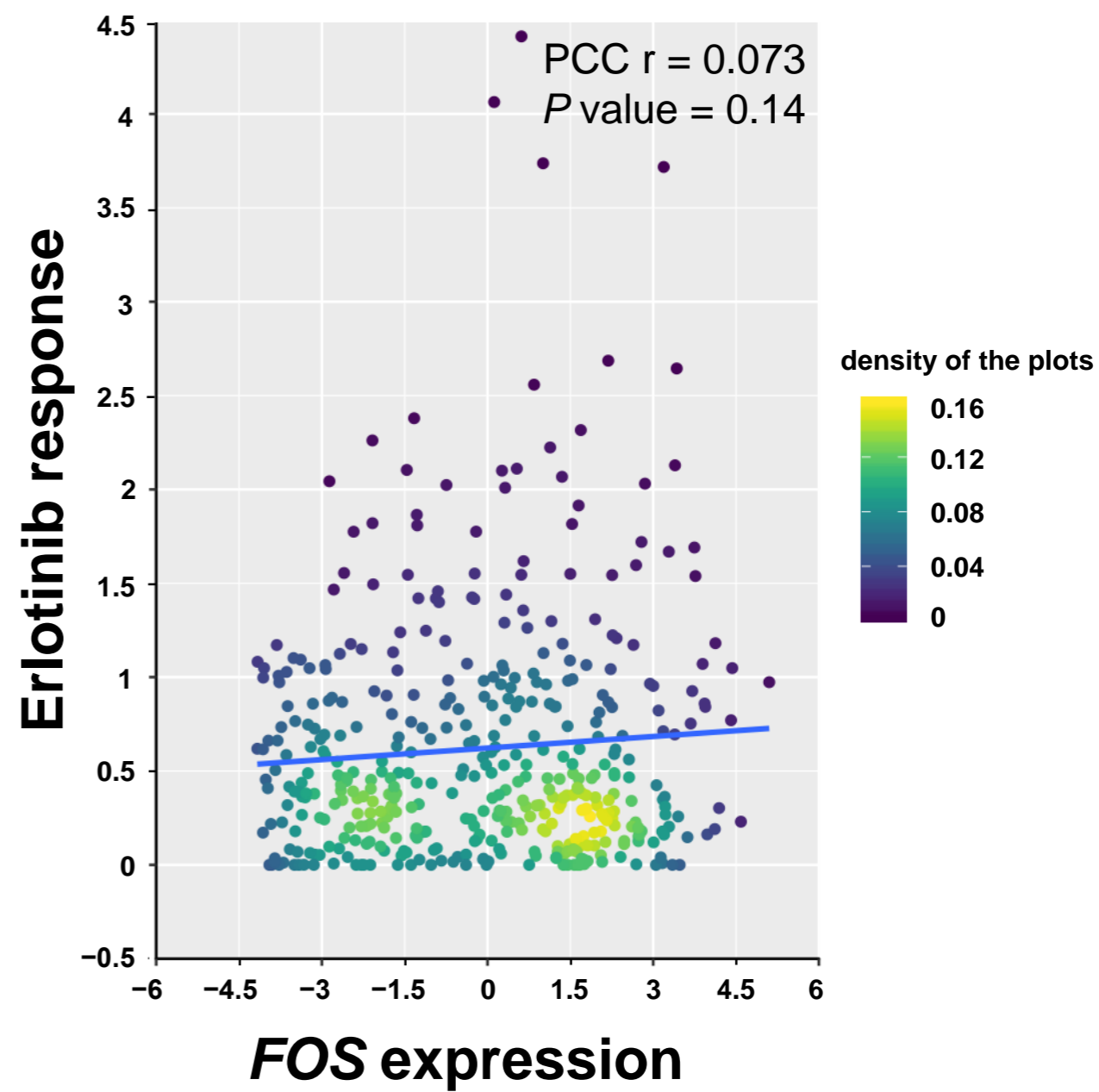

Supplement: Supplementary data 2 — Expression patterns of the known TRs related to erlotinib response. A. and B. Scatter plots showing the correlations between erlotinib response and mRNA levels of two transcriptional regulators (A) ZEB1 and (B) FOS. [file mmc2.pdf]

## Confounder correction

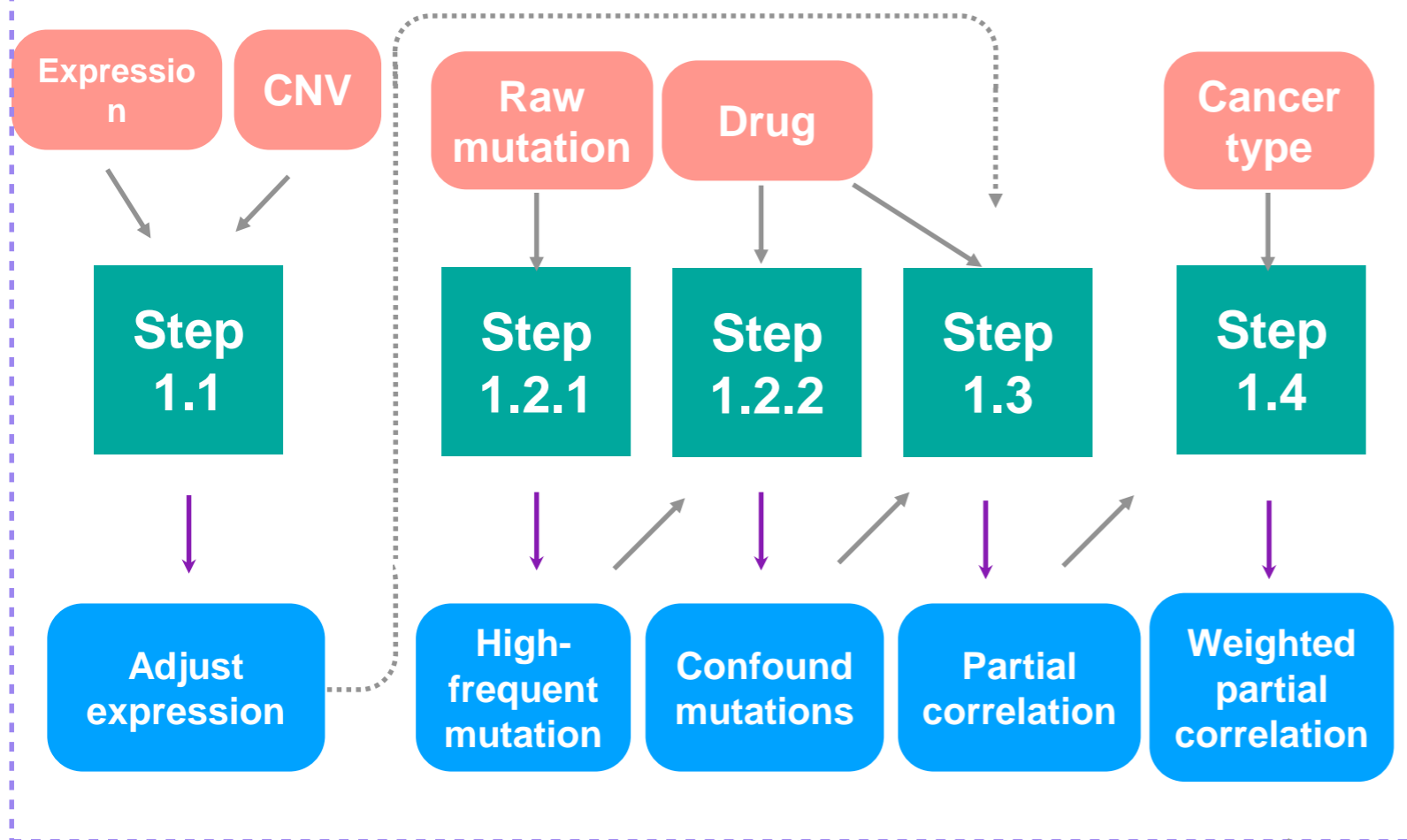

## Target inference

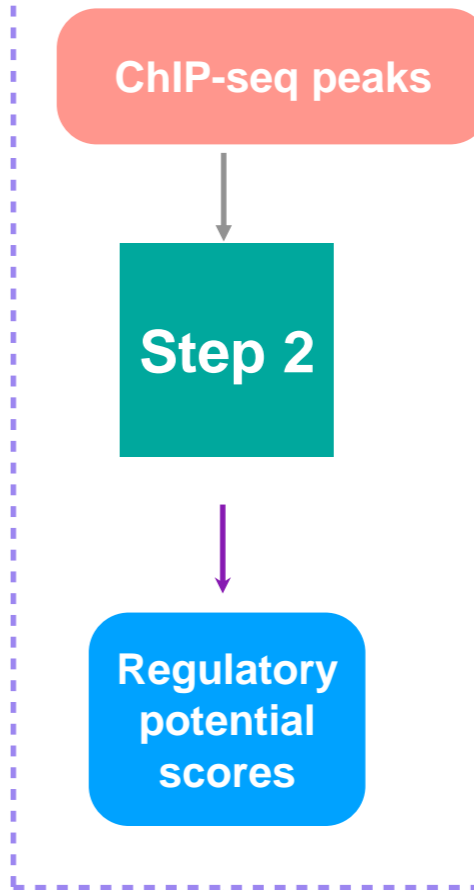

## TR identification

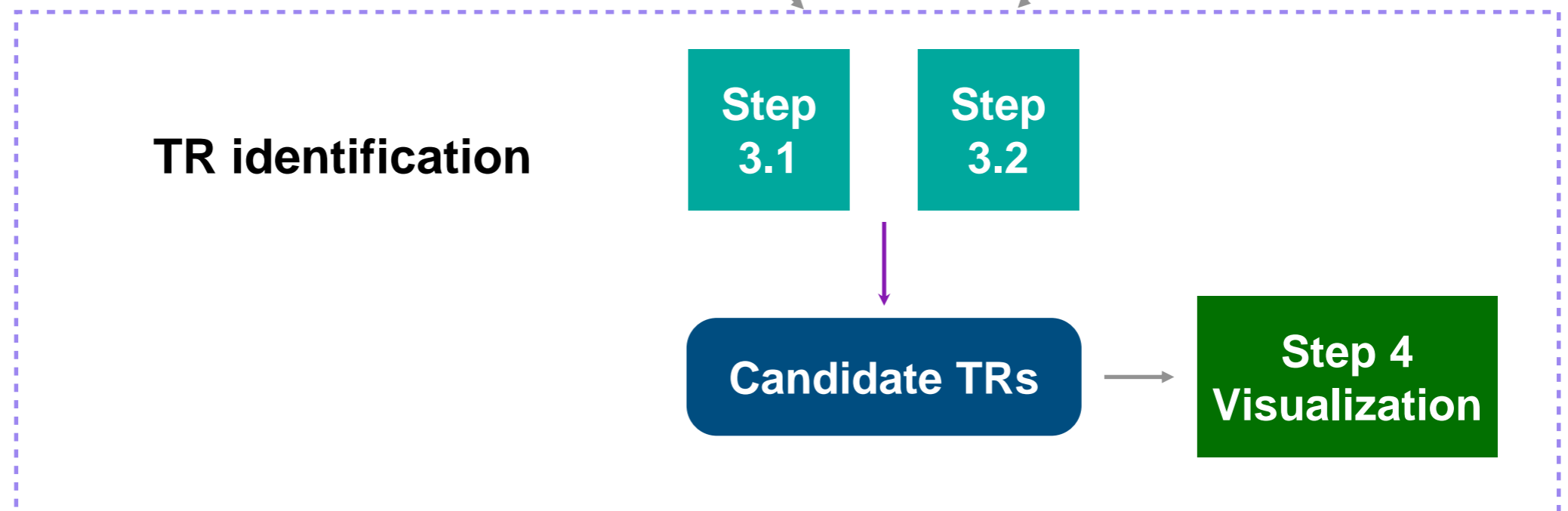

Supplement: Supplementary Figure S2 — Graphical representation of the RePhine workflow. The detailed graph showing the input data, output data, and all the steps. The pink rounded rectangles represent the raw input data. The blue rounded rectangles represent the intermediate output. The black rounded rectangle represents the final output of transcriptional regulators (TRs). The green rectangles represent the steps of the workflow corresponding to the method sections. The grey arrows represent the input and purple arrows represent the output. [file mmc3.pdf]

# A Instance

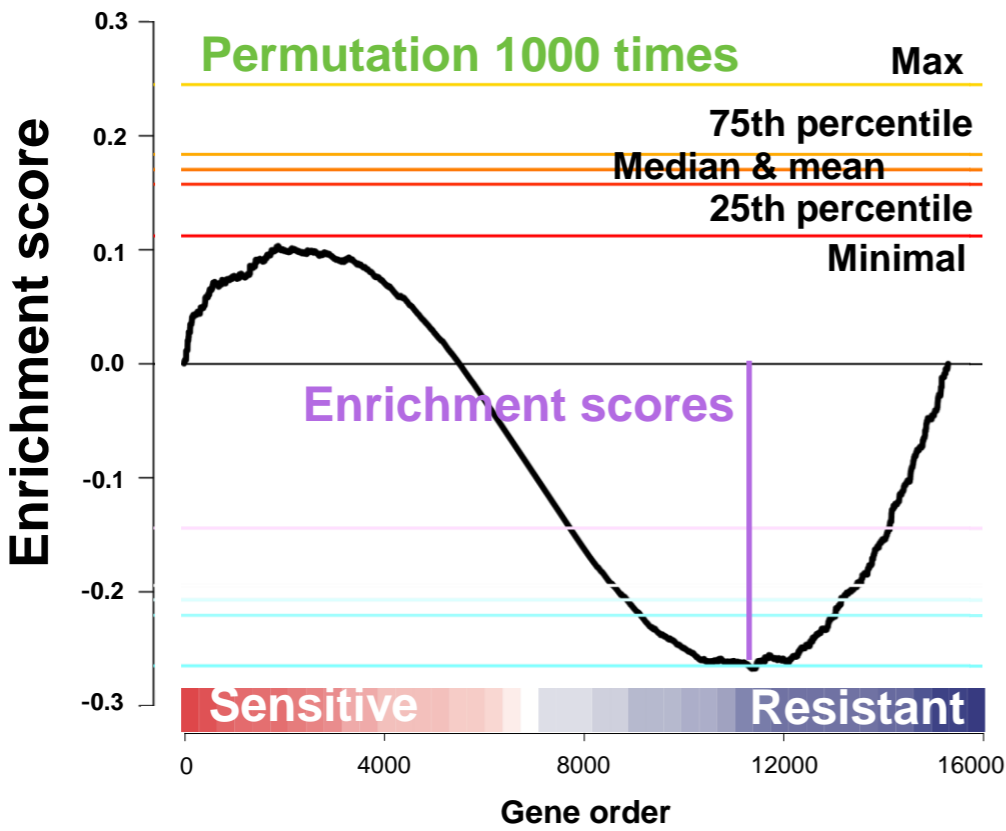

# B Negative enrichment

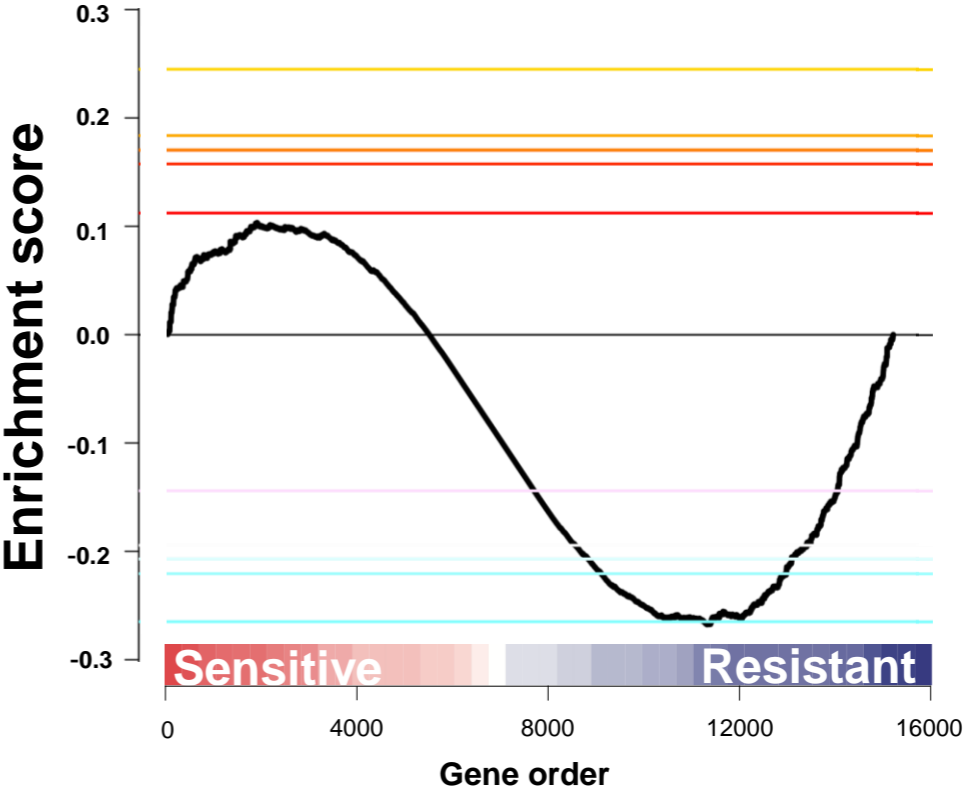

# C Positive enrichment

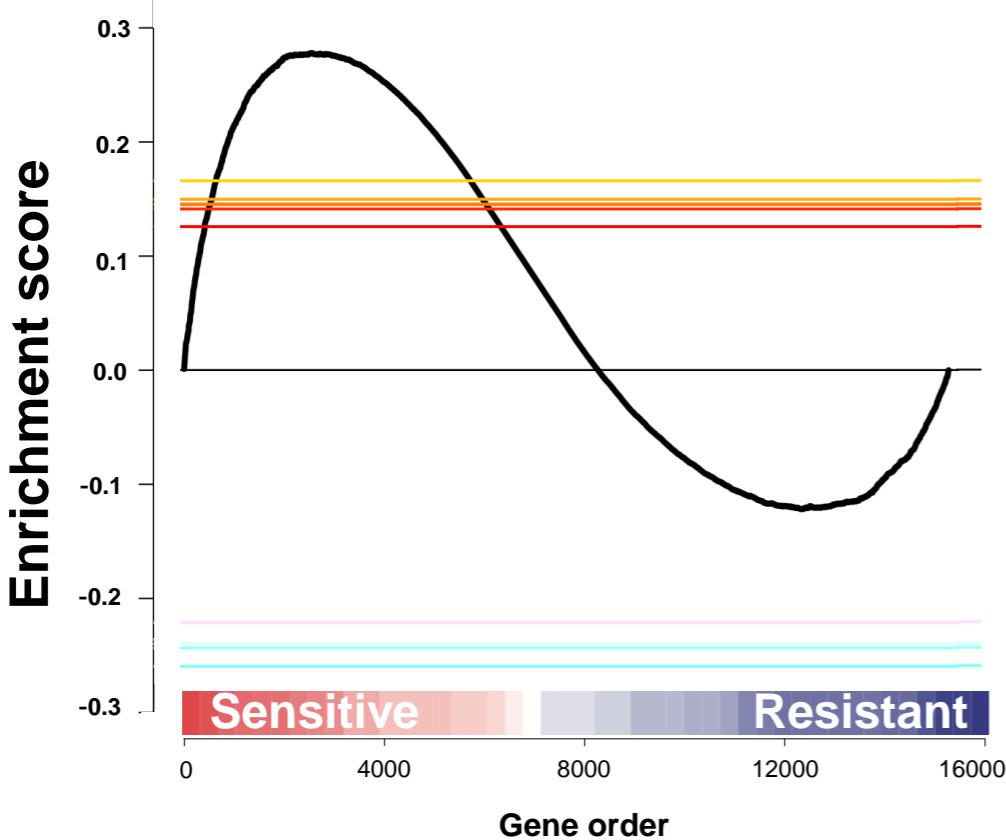

# D No enrichment

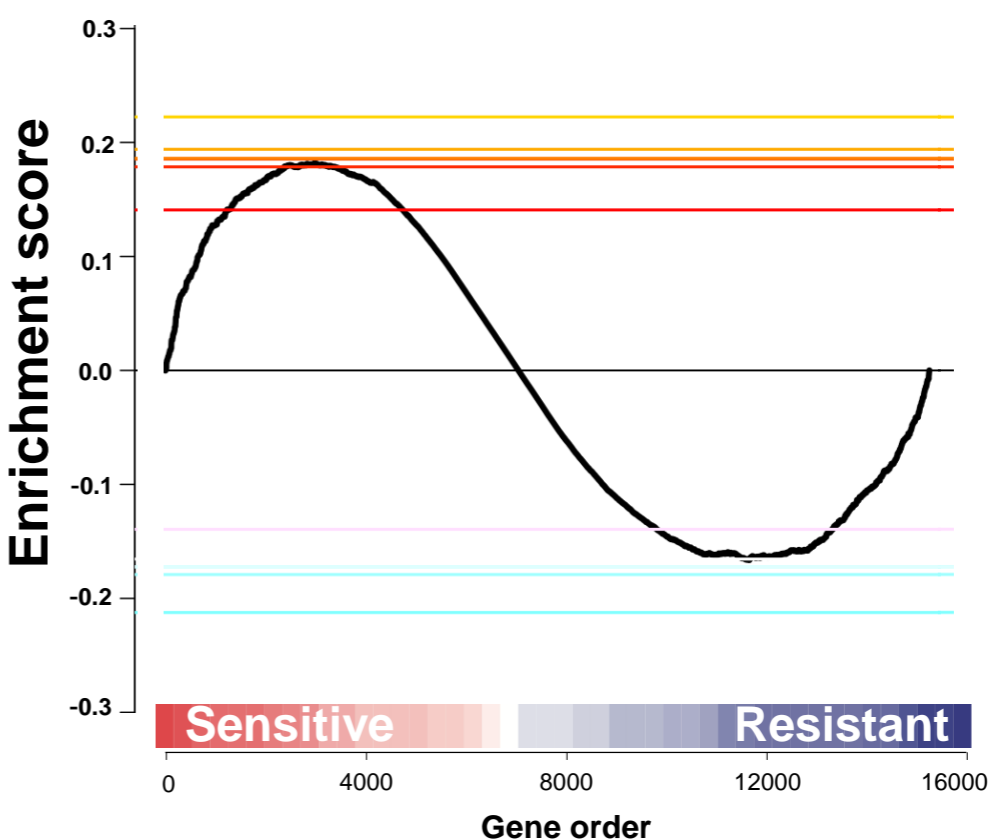

Supplement: Supplementary Figure S3 — Distinguishing patterns of TRs by examining whether TR targets have concordant partial correlations. A. Illustration of transcriptional regulator (TR) patterns. The targets are ordered by partial correlation coefficients. The accumulation of Dhit - Dmiss was then calculated (the Y-axis, details in methods). The enrichment score is determined by the maximum deviation from zero. Lines with warm colors or cool colors represent permutation distributions of the negative and positive enrichment scores receptively. B.–D. Three typical TR patterns with enrichment that is: negative (B), positive (C), and none (D). [file mmc4.pdf]

**A**

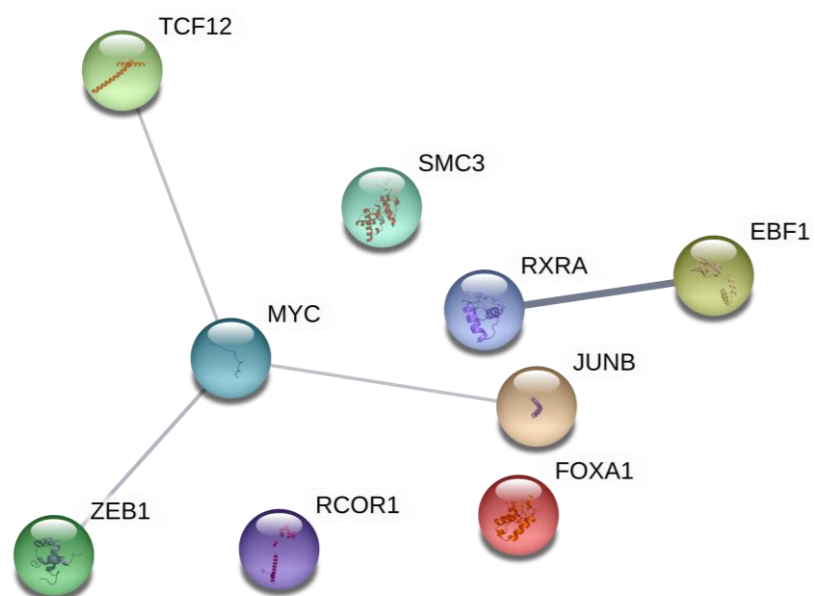

**Red TRs**

**B**

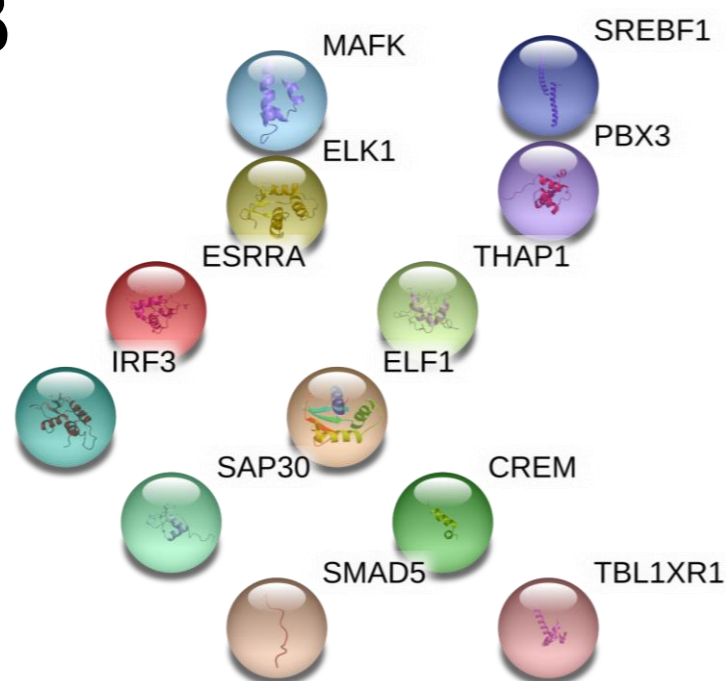

**Green TRs**

**C**

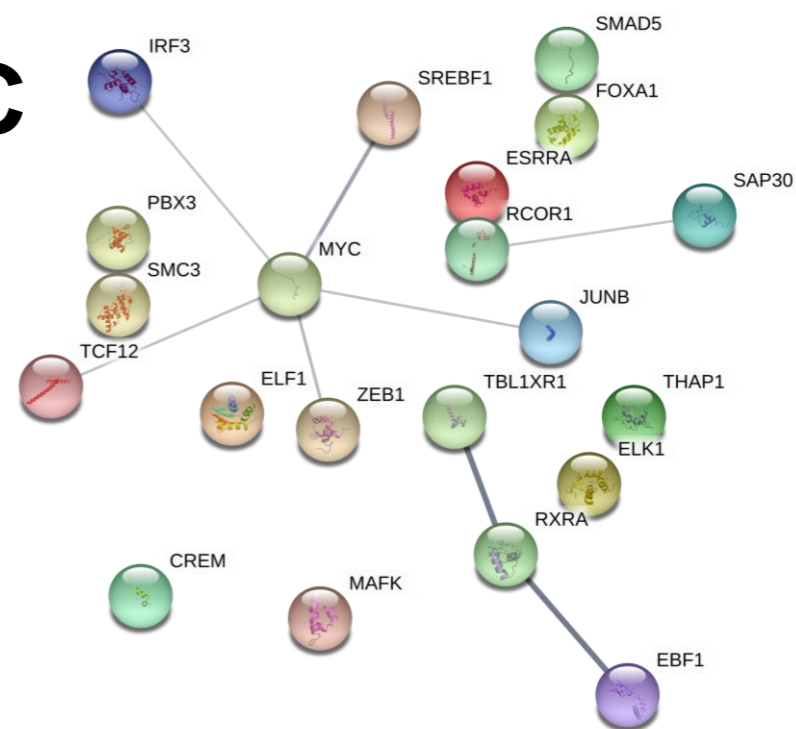

**Green + red TRs**

Supplement: Supplementary Figure S6 — PPI networks among candidate TRs from RePhine and correlation analysis. Panels A and B illustrated protein-protein interaction (PPI) networks among (A) CA-RePhine-shared-correlated transcriptional regulators (red TRs in Figure 2c) and (B) CA-correlated-only TRs (green TRs). C. PPI network by pooling the green and red TRs together. [file mmc7.pdf]

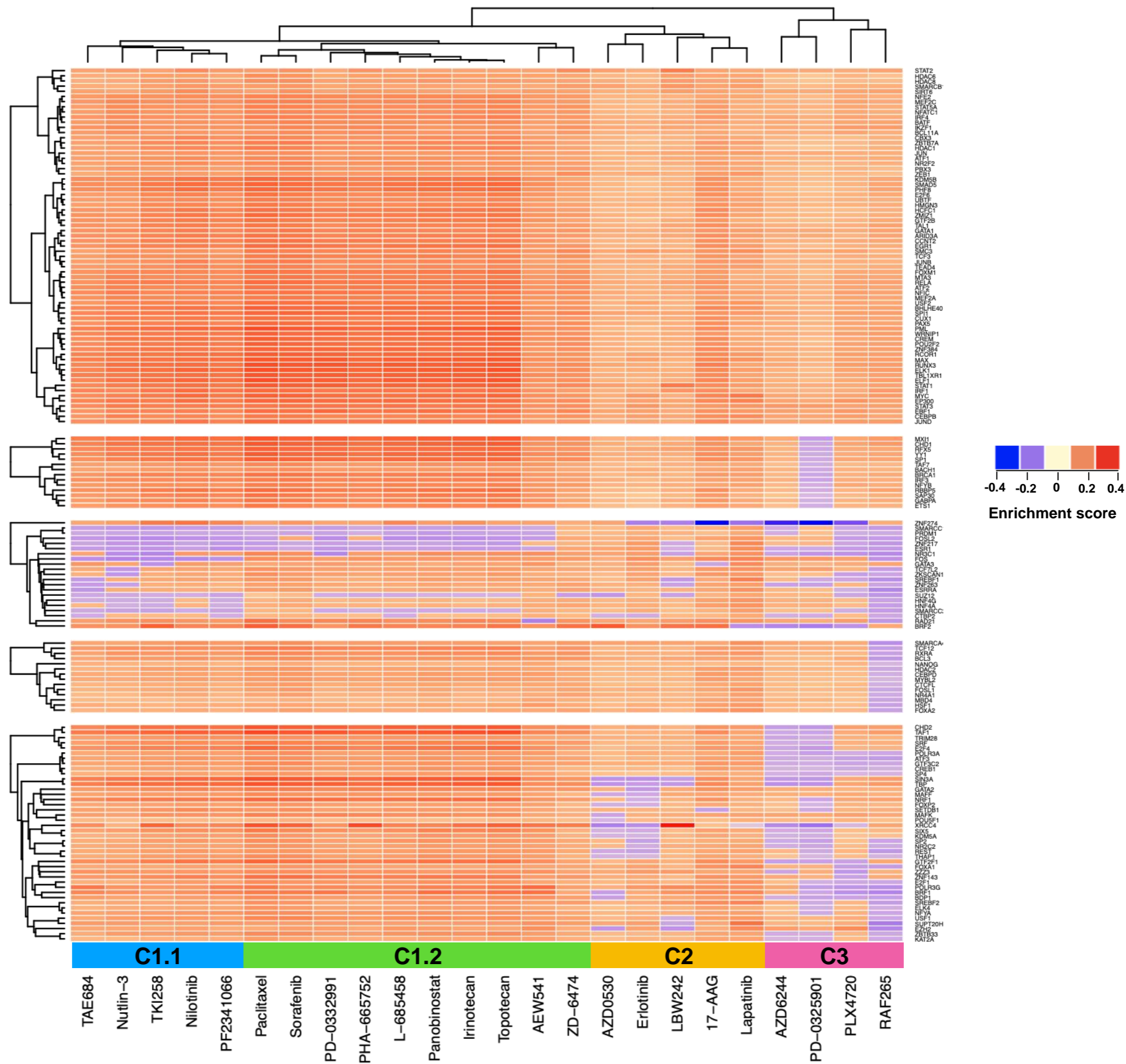

Supplement: Supplementary Figure S7 — Heatmap of all TR response signatures derived from GSEA enrichment scores (ES). Blue colors represent negative values of enrichment scores (ES) for transcriptional regulators (TRs) in the drug from gene set enrichment analysis (GSEA). Red colors represent positive values of ES. Color scale represents the degree of enrichment scores. Clusters are shown under the heatmap. [file mmc8.pdf]

A JHH-7 cells

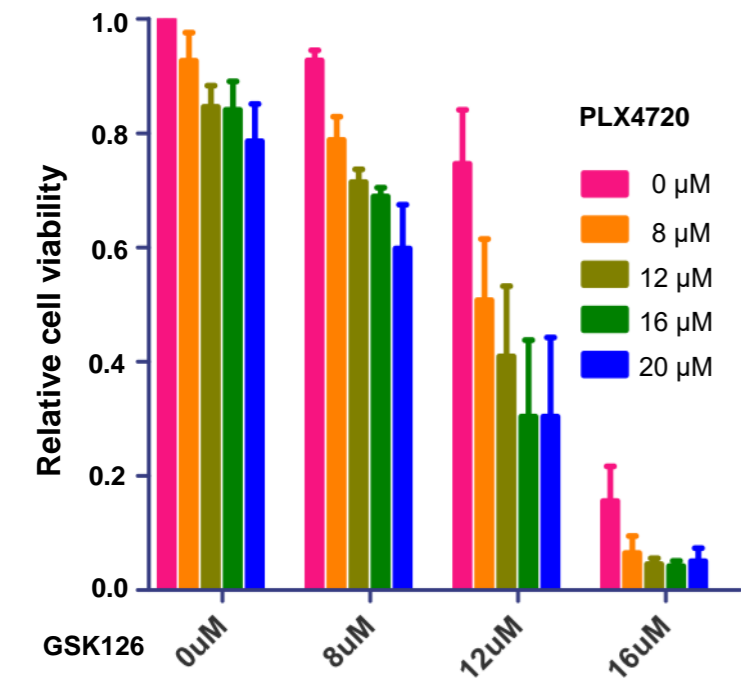

B

| PLX4720 ( $\mu$ M) | GSK126 ( $\mu$ M) | Fraction | CI    |
|--------------------|-------------------|----------|-------|
| 8                  | 8                 | 0.212    | 1.187 |
| 8                  | 12                | 0.492    | 1.087 |
| 8                  | 16                | 0.935    | 0.813 |
| 12                 | 8                 | 0.285    | 1.176 |
| 12                 | 12                | 0.591    | 1.041 |
| 12                 | 16                | 0.954    | 0.765 |
| 16                 | 8                 | 0.311    | 1.257 |
| 16                 | 12                | 0.696    | 0.963 |
| 16                 | 16                | 0.958    | 0.760 |
| 20                 | 8                 | 0.402    | 1.163 |
| 20                 | 12                | 0.696    | 1.000 |
| 20                 | 16                | 0.949    | 0.797 |

Average CI =  $1.021 \pm 0.186$

C

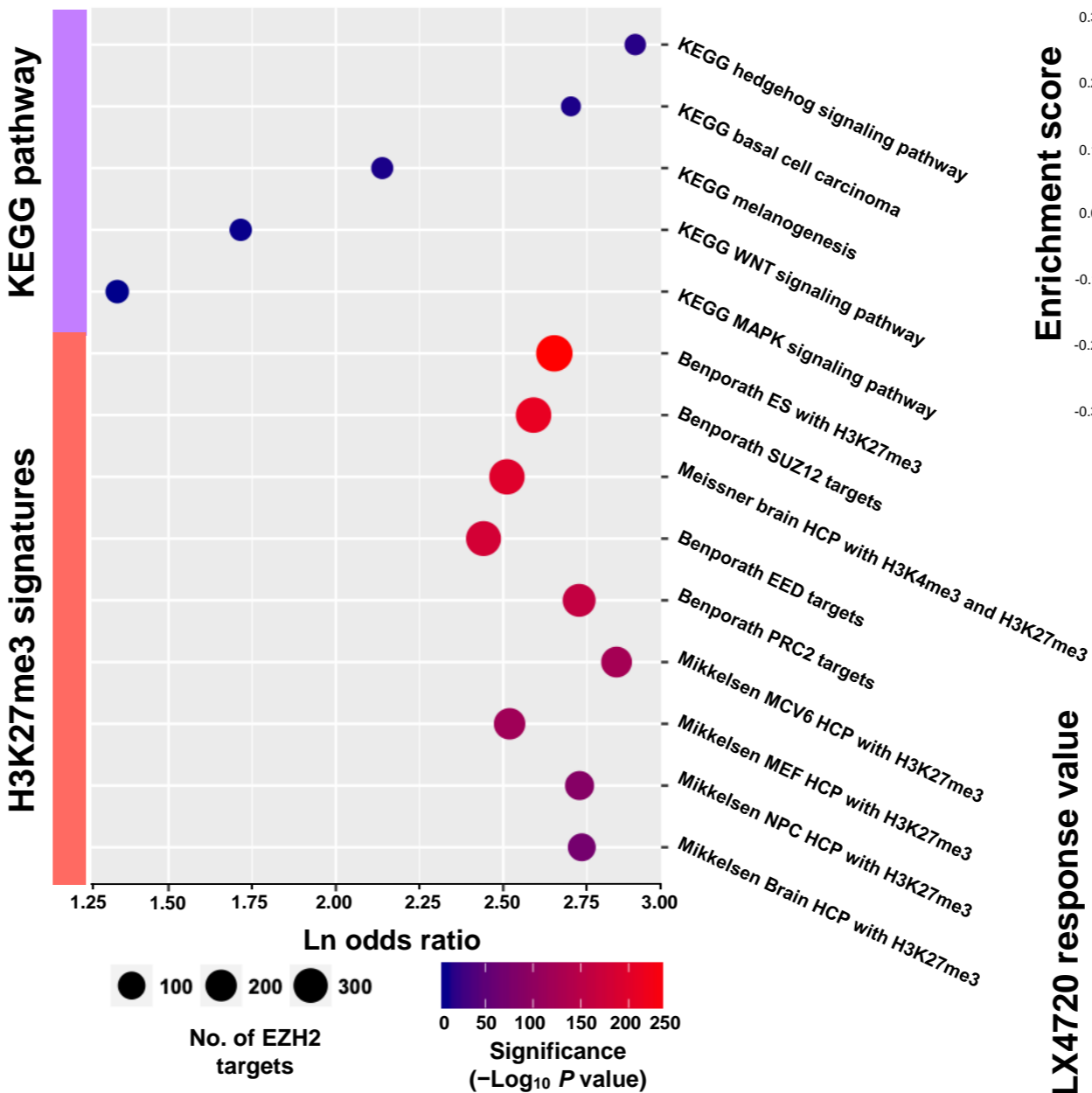

F

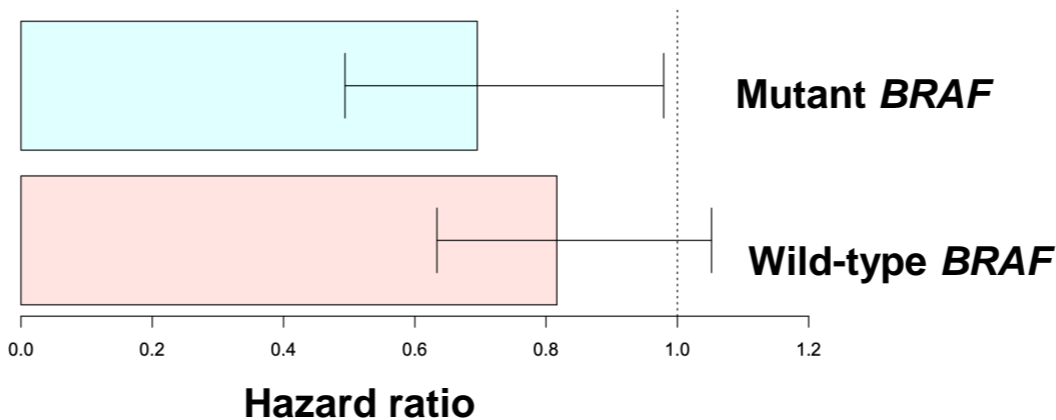

D ZNF217

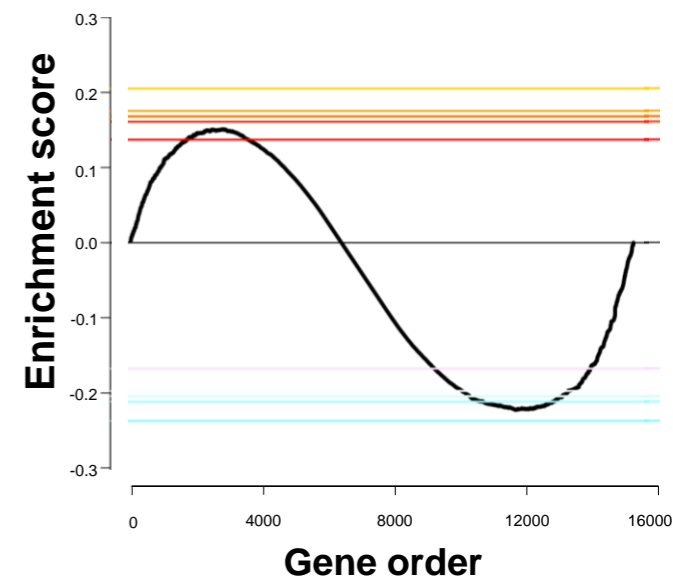

E

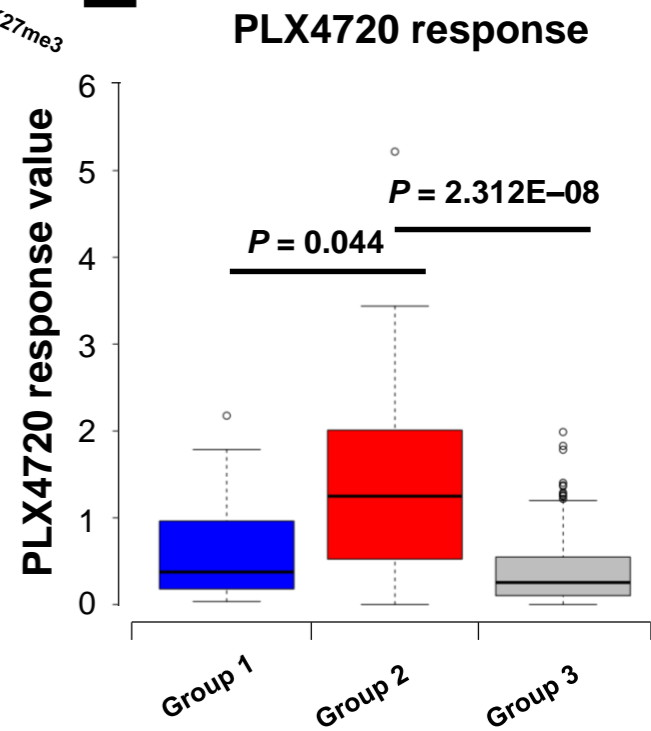

Supplement: Supplementary Figure S8 — EZH2 and H3K27me3-related genes are associated with BRAF inhibitor response. A. Bar plot for different concentration combinations of PLX4720 and GSK126 in a BRAF wild-type cell line JHH-7. Cell viability was normalized to untreated cells at 48h. B. CI for drug combinations at different dosages for JHH-7 cell line. Average CI (mean ± SD) was shown below. CI = 1 indicates additive effect; CI < 1 indicates synergistic effect; CI > 1 indicates antagonism. C. The over-represented KEGG pathways and H3K27me3 signatures of top 2000 EZH2 targets. D. Visualization of ZNF217 pattern. ZNF217 has a negative RePhine correlation to PLX4720. E. Distributions of PLX4720 response in three groups. Group 1, cell lines with mutations in both BRAF and H3K27me3-related genes; Group 2: cell lines only with mutated BRAF; Group 3: cell lines with both wild-type BRAF and H3K27me3-related genes. F. Bar plot for hazard ratios determined by the Cox regression model. Error bars represent 95% confidence interval. SKCM patients with higher PRC2 activity scores have lower hazard of death in BRAF mutant group (hazard ratio = 0.70, 95% confidence interval: 0.49–0.98, P = 0.0374). In contrast, in BRAF wild-type group, PRC2 activity scores were not significantly associated with hazard of death (hazard ratio = 0.82, 95% confidence interval: 0.63–1.05, P = 0.117). [file mmc9.pdf]

**A**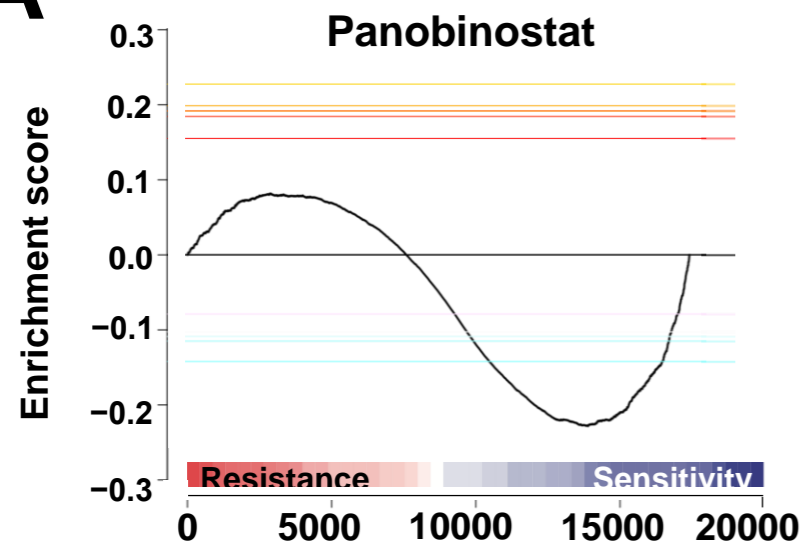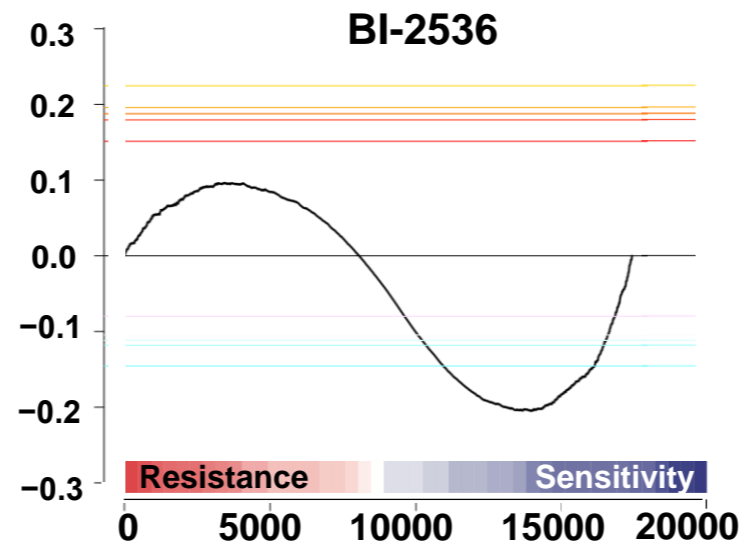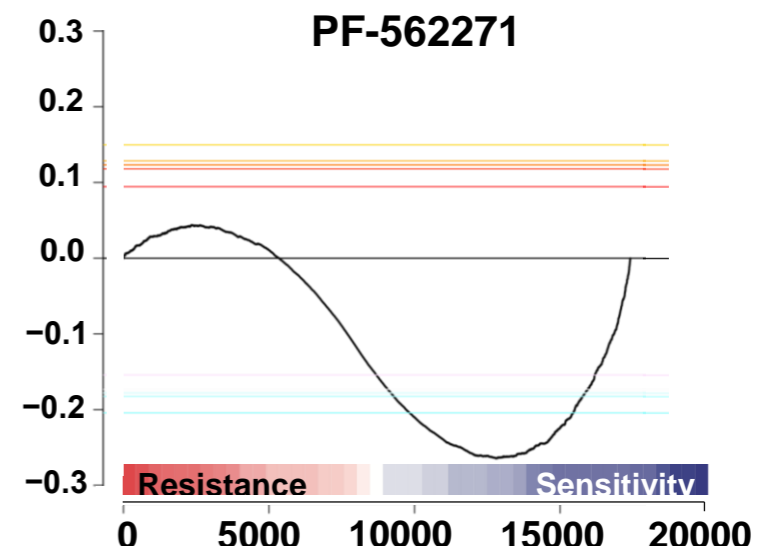**B**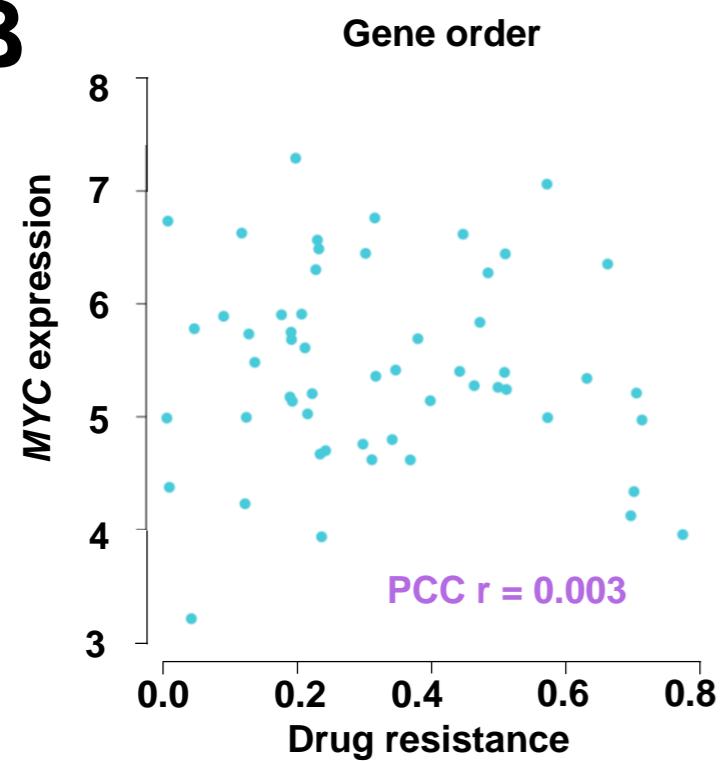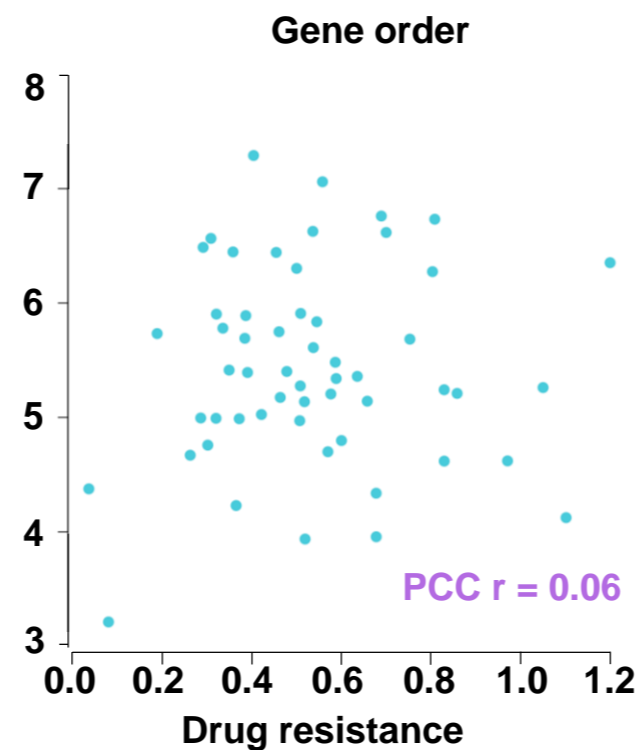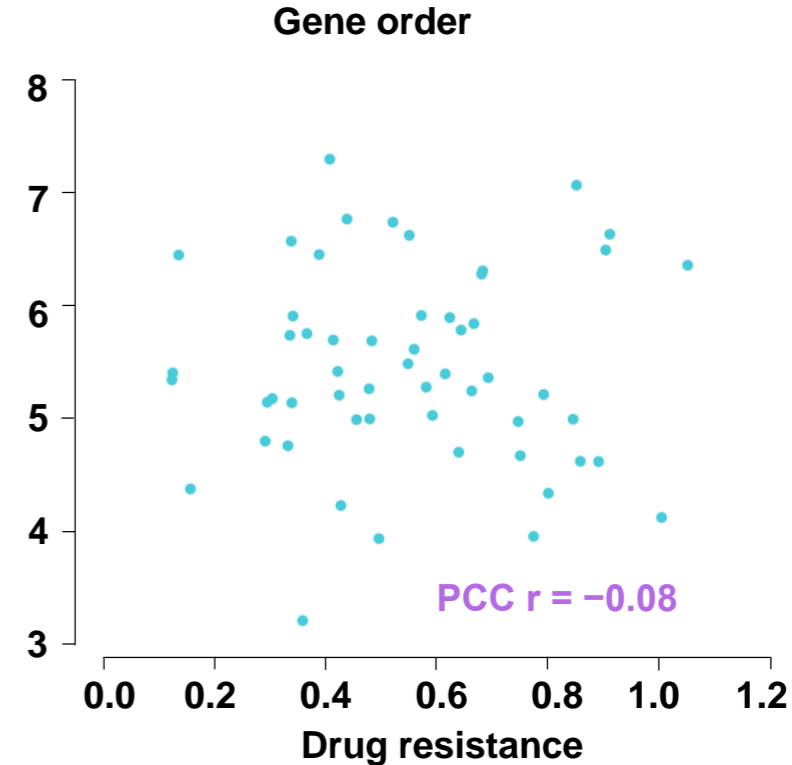**C**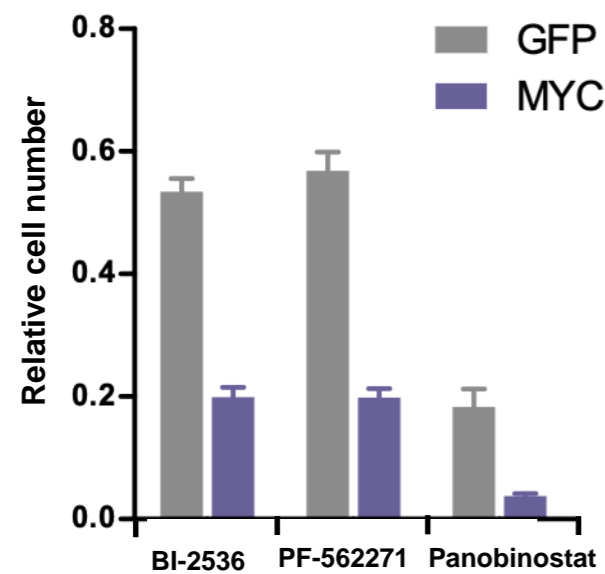

Supplement: Supplementary Figure S9 — Application of RePhine in an independent liver cancer dataset. A. Visualizations of RePhine-correlation of MYC in BI-2536, Panobinostat and PF-562271 respectively in liver cancer cell lines. B. Scatter plots showing the correlation between MYC expression and the drugs in order. There is no significant correlation exhibiting between MYC expression and drug response. C. Relative cell number under drugs treatment after MYC activation. The Y-axis is the relative cell number normalized to treatment with DMSO. Cell number decreasing after MYC activation indicates MYC sensitizes the cells to these drugs. GFP is the control without MYC activation. [file mmc10.pdf]
