# Supplementary material for: RePhine: An Integrative Method for Identification of Drug Response-related Transcriptional Regulators
Source: Genomics Proteomics Bioinformatics. 2021 Mar 10;19(4):534–48. doi: 10.1016/j.gpb.2019.09.008 (PMC9040019; doi:10.1016/j.gpb.2019.09.008)
Supplement: Supplementary Figure S4 — RePhine performance evaluation in simulated data. RePhine has an improved performance in comparison with correlation analysis, logistic model, and GSEA. The RePhine performance in the simulated data. Blue, green, yellow and brown bars represent correlation method “Corr”, logistic methods “Logistic”, gene set enrichment analysis (GSEA) and RePhine method respectively. The asterisk represents significance levels (*, P < 0.05; **, P < 0.01; ***, P < 0.001). In the scenario (A and B, F–H), the counts of the simulated positive (drug response-related) and negative (drug response-non-related) TRs are both 100. In the scenario (I) the counts of the simulated positive and negative TRs are 10 and 100 respectively. A. AUC of the ROC of all the methods. RePhine shows an improved performance in the scenario with the confounders (P = 9.083E−05, 3.389E−05, 0.02868 as RePhine vs. correlation analysis (CA), logistic and GSEA respectively; one-side paired t-test is used as follows). The assumed correlation coefficient is fixed to 0.15, low level of expression noise is added (level = 2) to synergize with the effect of the confounders. No noise is added to regulatory potential (RP scores). The target count is 500 (5% of all 10000 genes). B. RePhine has an improved performance than CA and logistic regression model with the noise of the expression (P = 0.002189 and 0.001749 as RePhine vs. CA and logistic respectively. The correlation coefficient is set to 0.3 (before the noise exposure), No noise is added to RP scores and the target count is 500. C. ROC of RePhine and CA with different correlation coefficients. Levels of the noises added to expression and RP scores (sampling from real data) are both 5 in this scenario. The target count is 500. D. the distribution of the noise added to RP scores derived from the standard deviations of the replicates of the ChIP-seq data. E and F. RePhine has the better performance than GSEA with the noise of RP scores following either of the distribution [file mmc5.pdf]

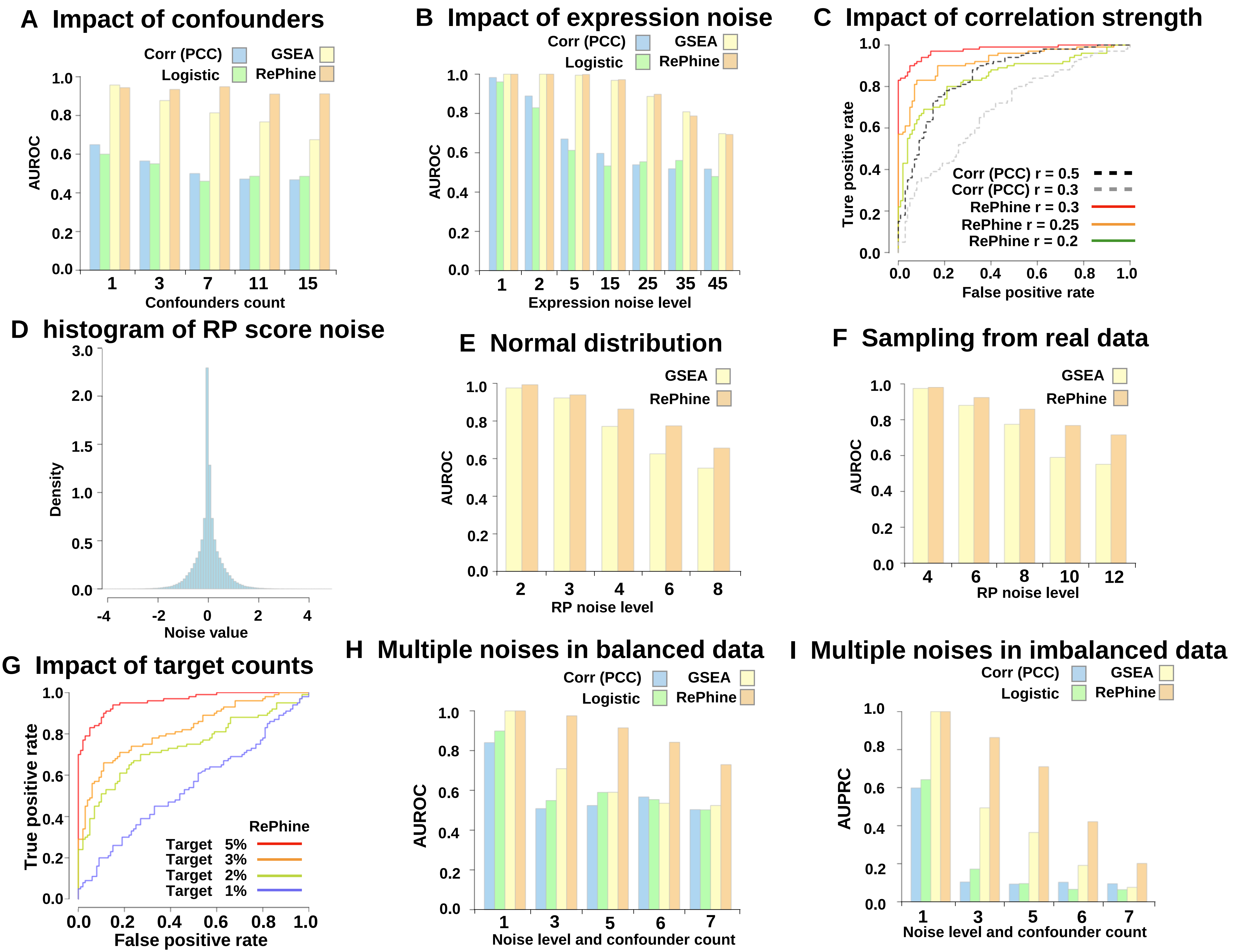
A Impact of confounders
Corr (PCC)
GSEA
Logistic
RePhine
AUROC
Confounders count
B Impact of expression noise
Expression noise level
C Impact of correlation strength
Ture positive rate
False positive rate
Corr (PCC) r = 0.5
Corr (PCC) r = 0.3
RePhine r = 0.3
RePhine r = 0.25
RePhine r = 0.2
D histogram of RP score noise
Density
Noise value
E Normal distribution
RP noise level
F Sampling from real data
G Impact of target counts
True positive rate
RePhine
Target 5%
Target 3%
Target 2%
Target 1%
H Multiple noises in balanced data
Noise level and confounder count
I Multiple noises in imbalanced data
AUPRC
