# Supplementary material for: RePhine: An Integrative Method for Identification of Drug Response-related Transcriptional Regulators
Source: Genomics Proteomics Bioinformatics. 2021 Mar 10;19(4):534–48. doi: 10.1016/j.gpb.2019.09.008 (PMC9040019; doi:10.1016/j.gpb.2019.09.008)
Supplement: Supplementary Figure S5 — Comparison of RePhine uniP and correlation coefficients between TR mRNA levels and drug response in CCLE dataset. A. Distributions of Spearman correlation coefficients between RePhine univariate P value (uniP, in log scale with the direction of sensitivity or resistance) and Pearson correlation coefficient between transcriptional regulator (TR) mRNA level and drug response in all 24 Cancer Cell Line Encyclopedia (CCLE) drugs illustrated by the violin plot. Each dot stands for the correlation coefficient between univariate P value (uniP) and coefficients of CA across all the TRs in the given drug. (B) and (C) are two example drugs among the 24 drugs and illustrated as the scatter plots for the significance (uniP) of TR-RePhine correlation (X-axis) and correlation coefficients of CA (Y-axis) respectively. B. Patterns of erlotinib. Each dot is a TR. C. Patterns of paclitaxel. D. top candidates with multivariate p-value (multiP) from RePhine results. Purple TRs are additionally within RePhine multivariate significance cutoffs (mulP < 0.005) but are not as significant in correlation analysis (CA). TRs that were not selected by the Elastic-Net model were in grey. The X-axis and Y-axis are the multiP and correlation P value (corP) on −Log10 scales respectively. [file mmc6.pdf]

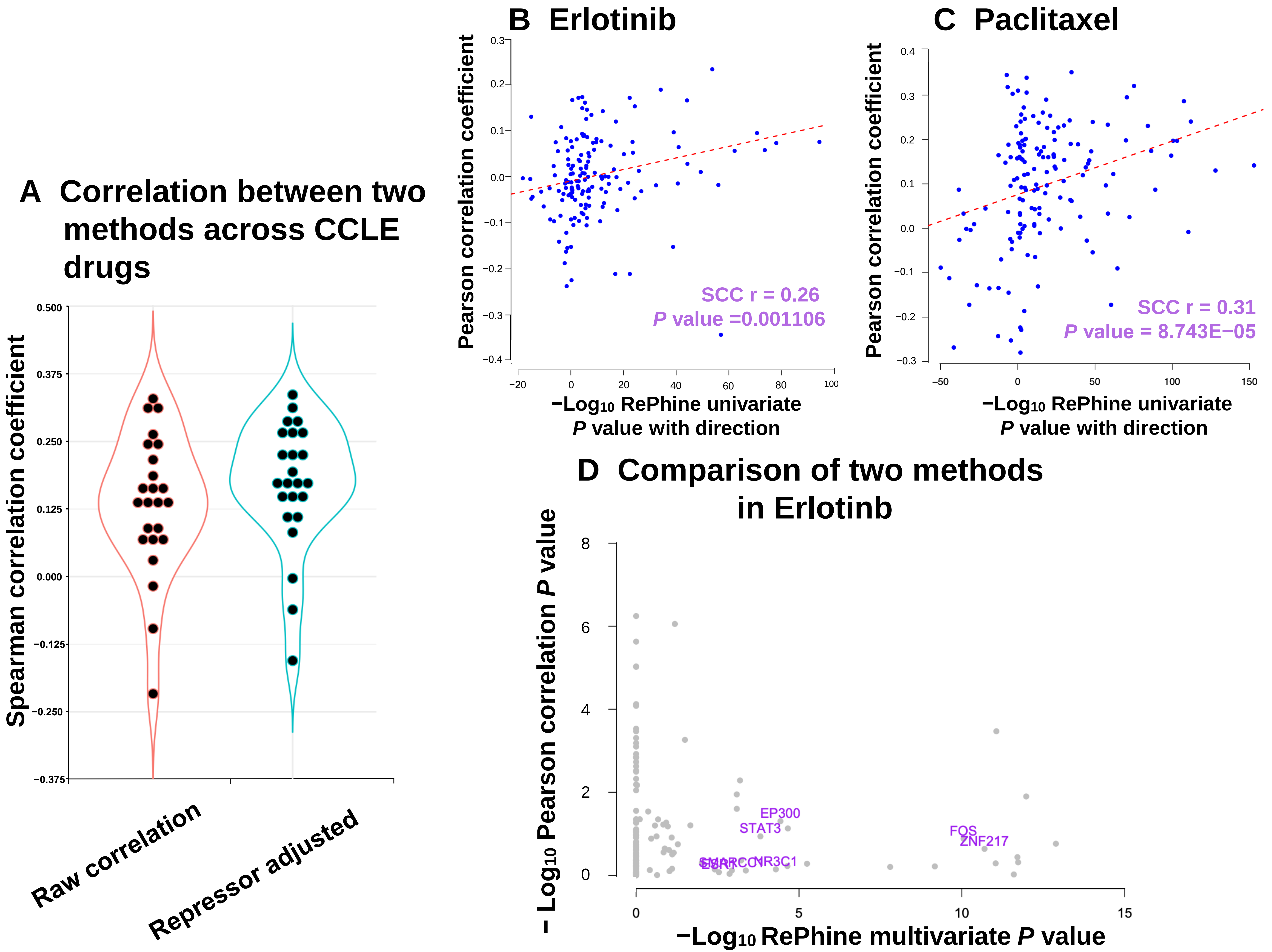
A Correlation between two methods across CCLE drugs
Spearman correlation coefficient
0.500
0.375
0.250
0.125
0.000
−0.125
−0.250
−0.375
Raw correlation
Repressor adjusted
B Erlotinib
Pearson correlation coefficient
SCC r = 0.26
P value =0.001106
−Log10 RePhine univariate
P value with direction
C Paclitaxel
Pearson correlation coefficient
SCC r = 0.31
P value = 8.743E−05
−Log10 RePhine univariate
P value with direction
D Comparison of two methods in Erlotinb
− Log10 Pearson correlation P value
EP300
STAT3
NR3C1
FOS
ZNF217
−Log10 RePhine multivariate P value
